# Pannexin-1 Is Blocked by Its C-Terminus through a Delocalized Non-Specific Interaction Surface

**DOI:** 10.1371/journal.pone.0099596

**Published:** 2014-06-09

**Authors:** Michelle Dourado, Evera Wong, David H. Hackos

**Affiliations:** Department of Neuroscience, Genentech, South San Francisco, California, United States of America; University of Bern, Switzerland

## Abstract

The Pannexin-1 (Panx1) channel is known to become activated under a variety of physiological conditions resulting in the release of medium-sized molecules such as ATP and amino acids from the cell. The detailed molecular mechanism of activation of the channel resulting in the opening of the Pannexin pore is poorly understood. The best-studied gating mechanism is caspase-3/7-mediated cleavage and truncation of the c-terminus. In the absence of caspase-cleavage, the c-terminal peptide maintains the channel in the closed state, possibly by directly plugging the pore from the intracellular side. We sought to understand in detail the part of the c-terminus necessary for this interaction by alanine-scanning and truncation mutagenesis of the c-terminal gating peptide. These experiments demonstrate that no single amino acid side-chain is necessary for this interaction. In fact, replacing blocks of 10–12 amino acids in different parts of the c-terminal peptide with alanines fails to disrupt the ability of the c-terminus to keep the channel closed. Surprisingly, even replacing the entire c-terminal gating peptide with a scrambled peptide of the same length maintains the interaction in some cases. Further analysis revealed that the interaction surface, while delocalized, is located within the amino-terminal two-thirds of the c-terminal peptide. Such a delocalized and potentially low-affinity interaction surface is allowed due to the high effective concentration of the c-terminal peptide near the inner vestibule of the pore and likely explains why this region is poorly conserved between species. This type of weak interaction with a tethered gating peptide may be required to maintain high-sensitivity to caspase-dependent activation.

## Introduction

The pannexins are large-pore ion channels distantly related by sequence to the invertebrate gap junction channels, the innexins, and unrelated by sequence to the connexins [1]–[5]. Despite such sequence homology to the innexins, pannexin channels appear not to form gap junctions in vertebrates but instead form large-pore plasma-membrane hemichannels [6]. While three pannexin family-members are known to exist in mammals, only Pannexin-1 (Panx1) has thus far been extensively studied. When activated, Panx1 forms a channel that is capable of allowing permeation of relatively large molecules including ATP, amino acids, and various DNA-binding dyes such as ethidium, Yo-Pro, and To-Pro [5]. Interestingly, despite the large diameter of the Panx1 channel pore, Panx1 appears to be selective for anions [7]–[8], though clearly large cations such as ethidium and Yo-Pro are also able to permeate the channel (but perhaps at a slower rate).

The gating mechanism of Panx1 has not been completely defined and is likely to be quite complex as Panx1 has been shown to be activated in a variety of seemingly distinct physiological settings [9]. In one case, mechanical shear stress and low O_2_ tension can activate Panx1 in erythrocytes [10], an observation that has been verified using erythrocytes from Panx1 KO mice [11], though it is not clear whether these stimuli directly or indirectly activate Panx1 channels. In another case, Panx1 can be activated in neurons under ischemic conditions (which has also been verified using Panx1 KO mice) [12]–[13]. Direct phosphorylation (possibly at Y308) by Src family kinases has recently been proposed to be required for such ischemia-dependent Panx1 activation in neurons [14]. P2X7-dependent activation of the inflammasome and subsequent IL-1β release in macrophages has been proposed to involve Panx1 activation, potentially through a direct interaction between Panx1 and P2X7 [15]. However, recent experiments using macrophages isolated from Panx1 KO mice have cast doubt on this role of Panx1 since activation of P2X7 in KO macrophages still leads to pore dilation (as measured by Yo-Pro influx) as well as inflammasome activation and IL-1β secretion [12], [16]–[18]. P2X7-dependent (as well as high K^+^-dependent) activation of Panx1 has been observed to occur in astrocytes [19]–[20], though contradictory results have been obtained using astrocytes from either Panx1 KO mice or Panx1/Panx2 dKO mice [12], [21]. GPCR-dependent activation of Panx1 has also been observed, but this work has thus far not been verified using Panx1 KO mice [22]–[23]. Finally, simple membrane depolarization has been shown to activate Panx1 channels, though it is not clear whether this mechanism of gating is physiologically relevant or due to a direct effect of membrane voltage on Panx1 [15].

Perhaps the best understood direct gating mechanism to date (at least at the molecular level) is Panx1 activation by direct caspase-3/7 cleavage of the c-terminus resulting in the removal of the last 45–50 amino acids (depending on species) [17], [24]–[25]. Gating by this mechanism is necessary for the release of ATP and possibly other small molecules during apoptosis, some of which may be used by macrophages as homing signals, aiding in the rapid clearance of apoptotic cells [17], [24], [26]. Mechanistically, the c-terminal peptide has been proposed to directly block the Panx1 pore by binding within the pore from the intracellular side of the channel [25]. This conclusion is based on the observation that the c-terminal cysteine residue (C426) is able to form a disulfide bond with a cysteine introduced within the extracellular side of the pore (F54C). In this case, cleavage near the caspase cleavage site is unable to open then channel until the disulfide bond is reduced, indicating that the c-terminus is able to bind within the pore and maintain the channel in a non-conducting state [25].

**Figure 1:**
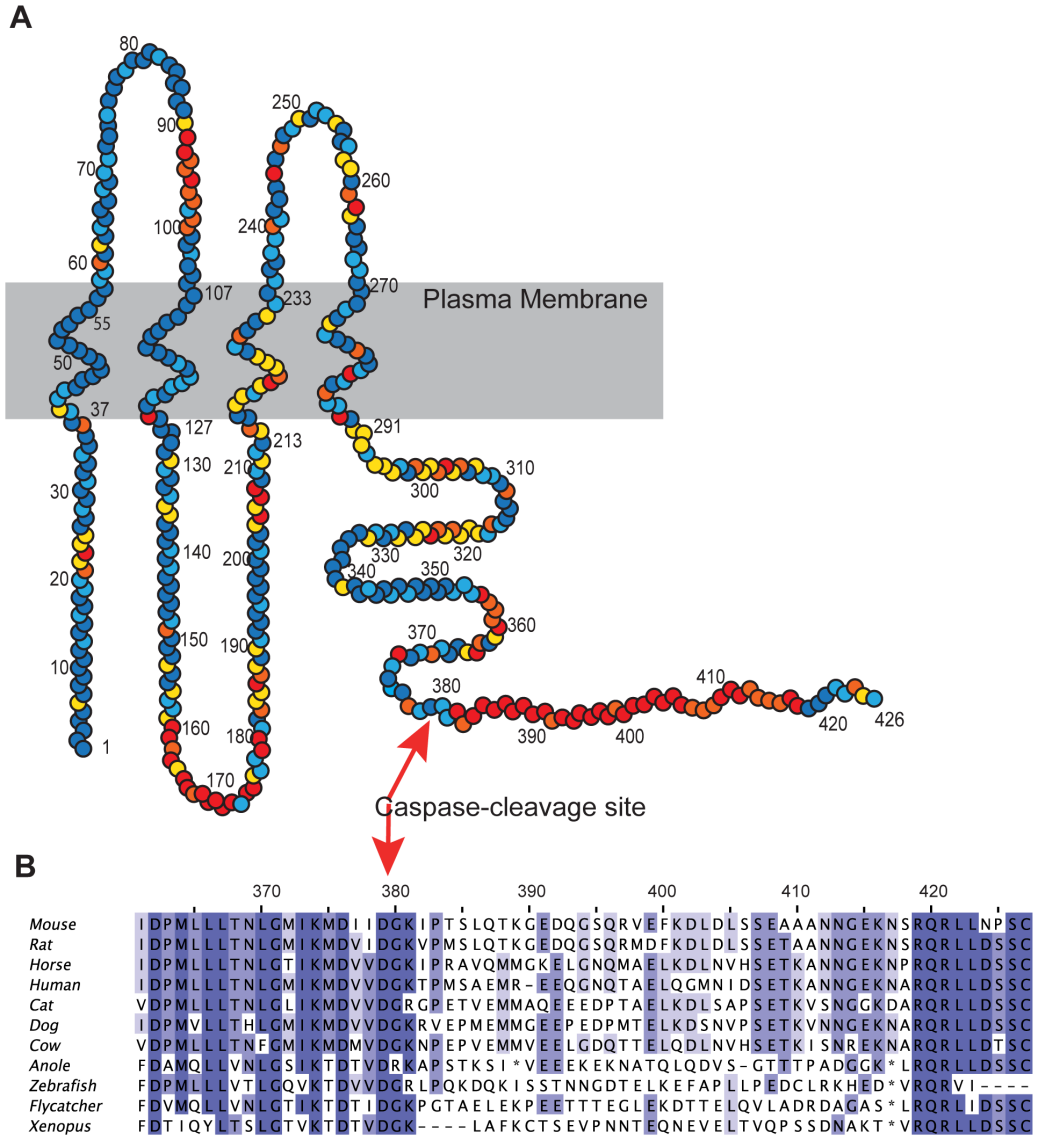
Sequence conservation between diverse vertebrate Panx1 channels. (**A**) Circles represent individual amino acids of mouse Panx1 from 1 to 426. Coloring represents sequence homology among a diverse sample of vertebrate Panx1 channel amino acid sequences. The sequences used in this analysis are mouse, rat, horse, human, cat, dog, cow, anole (*Anolis carolinensis*), zebrafish, collared flycatcher (*Ficedula albicollis*), and frog (*Xenopus tropicalis*). Colors were assigned as follows: dark blue (0–1 different residues present among these species), light blue (2 different residues), yellow (3 different residues), orange (4 differences), and red (5 or more different residues). Regions of low conservation among this sample of vertebrate species are part of the first extracellular loop (residues 90–100), part of the loop between TM2 and TM3 (residues 160–180), and the c-terminus after the caspase cleavage site (indicated). (**B**) A multiple sequence lineup shows the sequences of the c-terminus region in detail.

Interestingly, the c-terminal peptide is the least conserved region of the Panx1 channel between species (Figure 1). This is not expected since this region is thought to be critical for gating the channel. We sought to understand which sequence determinants within the c-terminus are important for its ability to prevent channel conductance with the hope of better understanding the interaction between the c-terminal peptide and the pore-forming part of the channel. Double alanine-scanning mutagenesis (by sequentially replacing pairs of residues in the c-terminus with alanines) reveals that no single residue side-chain is required for this interaction. Furthermore, replacing blocks of 10–12 residues with alanines indicates that no single 10-amino-acid region is necessary for the interaction. In fact, replacement of the entire proximal two-thirds of the c-terminus was required to disrupt the interaction, demonstrating a very large and delocalized interaction surface. In addition, fully scrambled c-terminal sequences were able to maintain the channel in the non-conducting state, indicating that extensive sequence variation is tolerated despite the critical nature of this region in gating. Thus, the length of the c-terminus appears to be more important than its amino-acid sequence for its ability to maintain Panx1 in the closed state. We propose that evolutionary pressure maintains this interaction between c-terminal gating peptide and the pore as low affinity in order to enhance the sensitivity to activation by caspase-3/7.

## Materials and Methods

### Ethics statement

Antibody generation in rabbits was carried out in compliance with a protocol approved by the Genentech Institutional Animal Care and Use Committee (IACUC). The IACUC has determined that this protocol results in no more than momentary or slight pain or distress, does not unnecessarily duplicate previous experiments, and is justified for this purpose, as there is no practical alternative. Once sufficient serum was obtained from the rabbits, animals were euthanized by intravenous injection with pentobarbital sodium.

### Electrophysiology

Panx1 current recordings were obtained from HEK293T (ATCC) cells heterologously expressing mouse Panx1. Briefly, cells were transfected 15–20 hours prior to recording using Lipofectamine LTX (Life Technologies) following the manufacturer's protocol. GFP was co-transfected at a 1∶10 ratio to aide in the identification of transfected cells. Whole-cell patch clamp recordings were obtained using a Molecular Devices Axopatch 200B patch clamp amplifier. Currents were measured by application of a voltage ramp from −100 mV to +100 mV using an extracellular recording solution containing (in mM): 155 NaCl, 3 KCl, 1 MgCl_2_, 1.5 CaCl_2_, 10 HEPES/NaOH, pH 7.4. The recording pipet intracellular solution contained (in mM) 120 CsF, 10 NaCl, 2 MgCl2, 10 HEPES/CsOH pH 7.2. Carbenoxolone was added at 100 µM to fully block Panx1 channels [27] for the purpose of leak subtraction.

### Molecular biology

Mouse and human Panx1 cDNAs were generated by direct DNA synthesis (Blue Heron Biotech) and subcloned into pCDNA3.1_Hygro (Invitrogen). Point mutations were made using the QuikChange Lightning site-directed mutagenesis kit (Agilent Technologies) as per manufacturer's instructions. Scrambled c-terminal peptide sequences where designed using the Shuffleseq application which is available online as part of EMBL's EMBOSS open bioinformatics software suite [28]. Scrambled cDNAs and cDNAs with larger block mutations were generated by direct synthesis (Blue Heron Biotech).

### Immunohistochemistry

HEK293T cells were plated onto 8-well Millicell EZ slides (Millipore) and transiently transfected with various Panx1 mutants. At 20 hours post transfection, cells were fixed, permeabilized, and stained with one of two affinity-purified rabbit polyclonal antibodies that bind to epitopes present in the mouse Panx1 c-terminus: YZ2865 or YZ2868. These antibodies were generated at YenZym Antibodies, LLC by injecting rabbits with keyhole limpet hemocyanin (KLH)-conjugated peptides with the following sequences: YZ2865 (CQRVEFKDLDLSSEAA) or YZ2868 (CANNGEKNSRQRLLNPS).

### Western blots

Surface expression of the various Panx1 mutants in this study was determined using the EZ-link Sulfo-NHS Biotinylation kit (Thermo Scientific). Cells were transfected 15–20 hours prior to surface biotinylation. Each set of experiments included 3 controls: mock transfection (pCDNA3.1 alone), full length Panx1 (FL), and Panx1 truncated at the caspase-cleavage site (Δ379). Briefly, transfected cells were washed with PBS and incubated with Sulfo-NHS-biotin solution in PBS at 4°C with rocking for 30 minutes. Quenching solution was added to each well to stop the reaction. Cells were then scraped into tubes and washed 3 times with Tris buffered saline to get rid of any remaining biotin. Cell lysis was carried out overnight at 4°C in Tris buffered saline containing 1% n-Dodecyl-beta-D-maltoside and protease inhibitor cocktail. The surface fraction was collected using Neutravidin bound agarose beads and eluted from the beads using gel loading buffer containing 4% SDS, 4% urea, 100 mM BME, 200 mM DTT. While it is possible that biotin may label intracellular protein by entering the cells through active Panx1 channels, in our hands, no difference in labeling was observed when cells were treated with the biotinylation reagent in the presence or absence of 50 µM carbenoxolone (see Figure S2). Nitrocellulose blots were incubated in Tris buffered saline containing 0.1% Tween and 1% BSA and 0.5 microgram/mL rabbit polyclonal anti-Pannexin-1 (mid) antibody (Invitrogen), followed by ECL HRP-linked anti-Rabbit secondary antibody (GE Healthcare, UK). To visualize the loading control, blots were subsequently labeled with anti-actin (cytoplasmic fraction) and anti-transferrin receptor (membrane fraction) using purified mouse anti-actin Ab-5 (BD Biosciences) and monoclonal anti-human transferrin receptor antibody (Invitrogen).

### Yo-Pro influx measurements

Yo-Pro influx measurements for measuring Panx1 channel activity were done as follows. Cells were transfected with full-length Panx1 or Panx1 mutant channels 15–20 hours prior to recording. Each 96 well plate contained mock transfected, full-length Panx1 (FL), and truncated Panx1 (Δ379) controls. Yo-Pro influx for all variants on the plate were normalized to the Δ379 control on the same plate to control for day-to-day variability in channel expression. At the time of recording, cell media was removed and replaced with Hank's Buffered Saline Solution (HBSS) with calcium and magnesium (Invitrogen) containing 1 µM Yo-Pro-1 at 37°C. Fluorescence images were collected every 60 sec over a period of 1 hour using a 10X Plan Fluor objective. The microscope was equipped with an environmental control system allowing us to maintain high humidity, 5% CO_2_ and 37°C throughout the imaging session. A Nikon Perfect Focus system (PFS) was used to allow imaging of up to 10 wells simultaneously. Fluorescence intensity was recorded and calculated using Nikon's NIS Elements software, which enables region-of-interest selection and automatic tracking of 20–25 nuclei per image at each time-point throughout the recording time. Graphs showing Yo-Pro fluorescence over time have error bars that represent the standard error of the mean (SEM) representing the variability in fluorescence among the 20–25 cells recorded from each well. Each mutant was measured at least 3 times on separate days and the combined results are presented as normalized slopes with error bars displayed as SEM with N representing the number of separate experiments.

### Multiple sequence alignment

Jalview version 2 was used for sequence alignment [29].

## Results

**Figure 2:**
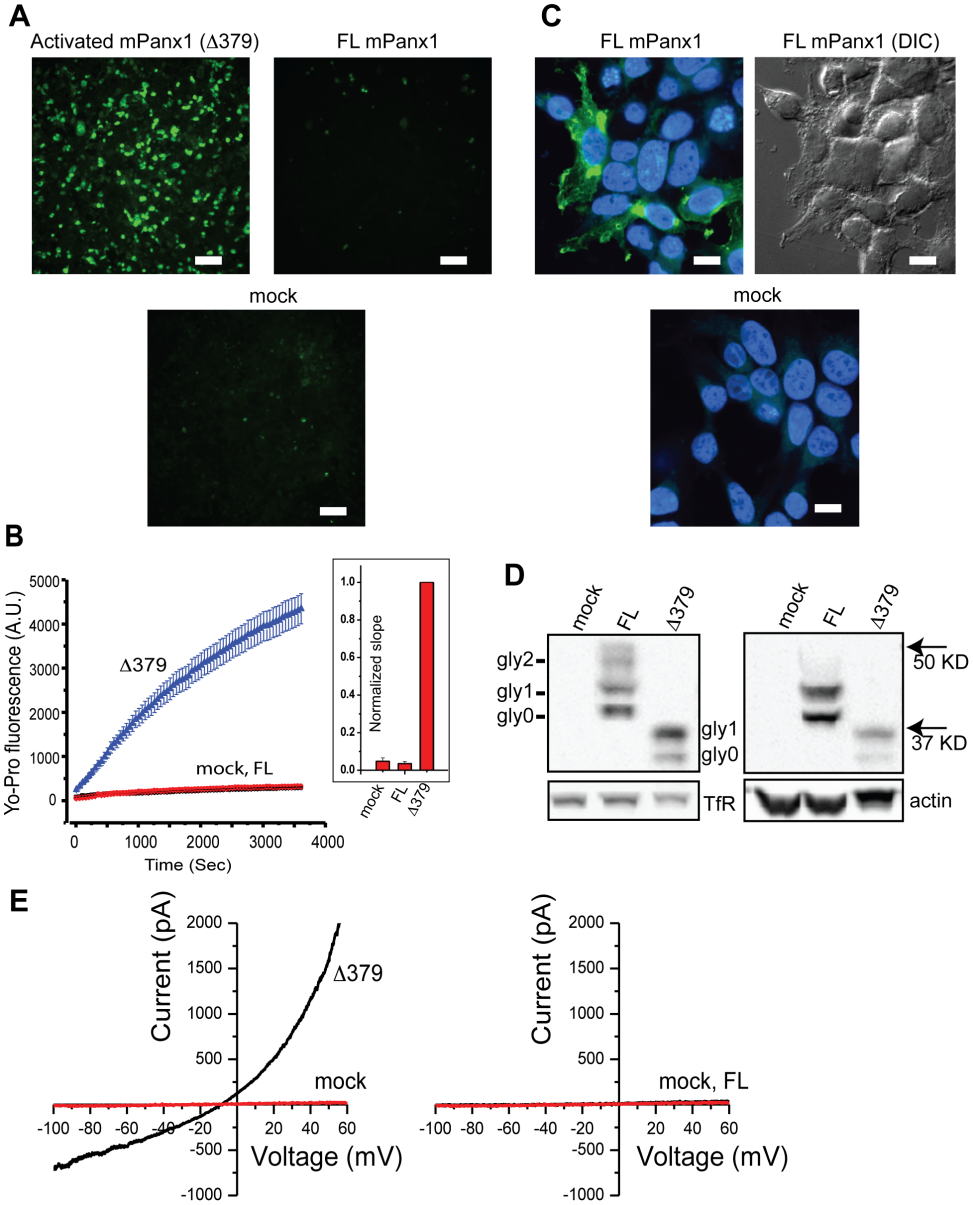
Unlike full-length mouse Panx1, Panx1 truncated at amino acid 379 (Δ379) is constitutively active. (**A**) HEK293T cells transfected with Δ379, full-length (FL) Panx1, or pCDNA3.1 vector alone (mock) were assayed for Yo-Pro influx (example images shown). Scale bars are 100 µm. (**B**) Time-lapse fluorescence microscopy was used to collect images of the Yo-Pro fluorescence once per minute over a period of 1 hour following addition of 1 µM Yo-Pro-1 to the cells. (**B-inset**) The functional activity of the channels is represented as the slope of the Yo-Pro uptake over the initial 30 minutes. A one-way ANOVA comparing all pairs of mean slopes with Tukey-Kramer correction for multiple comparisons was used to show that Δ379 has significantly higher Yo-Pro influx compared to mock and FL (p<0.01), while FL was not different than mock. Full-length and Δ379 Panx1 are expressed on the plasma membrane as determined by (**C**) ICC and (**D**) surface biotinylation-Western blot. A differential interference contrast (DIC) image (**C-right**) is shown side-by-side with the ICC of FL Panx1. Scale bars for ICC are 10 µm. Both the isolated (**D-left**) surface fraction as well as the (**D-right**) cytoplasmic fraction are shown with loading controls transferrin receptor (TfR) and actin respectively. Such Western blots show the three forms of full-length Panx1 (non-glycosylated gly0, partially-glycosylated gly1, and fully glycosylated gly2 as indicated) while only gly0 and gly1 forms are present in the case of Δ379. Typical whole-cell patch clamp recordings of cells expressing either (**E-left, black**) Δ379 or (**E-right, black**) FL Panx1 relative to empty plasmid (red in both left and right) are shown following leak subtraction with 100 µM carbenoxolone. The current-voltage (I–V) curves shown here were generated by ramping the membrane voltage from −100 mV to +60 mV in 500 msec (from a holding voltage of −20 mV).

In order to investigate the properties of caspase-3-activated mouse Pannexin-1 (Panx1), we generated constructs expressing either full-length (FL) Panx1 or Panx1 truncated at the caspase-3 cleavage site (Δ379) by specifically introducing a stop codon immediately following D379. Truncation after D379 produces the equivalent of a capsase-3-cleaved Panx1 channel without requiring activated caspase-3 enzyme, which could negatively affect the health of the cells. Expressed transiently in HEK293T cells, Δ379 showed robust channel activity as revealed by Yo-Pro influx following addition of 1 µM Yo-Pro-1 for 1 hour to the cells (Figure 2A). The full-length Panx1 channel showed no functional activity in this assay. Yo-Pro influx is Panx1-dependent since cells transfected with pCDNA3.1 alone showed no Yo-Pro fluorescence after 1 hour (Figure 2A).

To quantify this signal, we recorded Yo-Pro fluorescence over 1 hour at 1 min. intervals. Cells were co-transfected with a plasmid containing dsRed as a marker to identify which cells were transfected. Expression of Δ379 appeared to reduce the expression level of dsRed substantially, so in this case transfected cells were identified by the Yo-Pro influx activity itself. The rate of Yo-Pro influx was quantified by averaging the fluorescence of 25 individual transfected cells over time following Yo-Pro addition (Figure 2B). We repeated each set of experiments at least 3 times on separate days. For each set of experiments, we measured the initial slope of the Yo-Pro influx curve by fitting a line to this data (using the first 30 min.) and normalized the results relative to the Δ379 control for each dataset. The resulting normalized slope values were averaged and used as a quantification of constitutive channel activity (Figure 2B inset). We found that Δ379 but not full-length Panx1 nor mock-transfected cells showed significant Yo-Pro influx over the time-course of 1 hour. Since lack of Yo-Pro influx by full-length Panx1 could be due to lack of expression, we sought to verify membrane surface expression by immunocytochemistry (Figure 2C) and Western blotting (Figure 2D). By immunocytochemistry (ICC), the full-length channel appears to be robustly expressed on the membrane surface, though it is not possible by ICC alone to eliminate the possibility that Panx1 might be localized just inside the membrane and not actually on the surface. We were not able to use ICC to verify the membrane expression of Δ379 since the antibodies we use for ICC bind to c-terminal epitopes, which are absent in this c-terminal-truncated mutant channel, though clearly Δ379 is expressed sufficiently on the surface to allow robust Yo-Pro influx. Western blotting using an antibody that binds to the membrane-spanning part of Panx1 confirmed membrane expression of both Δ379 and full-length Panx1. To demonstrate channel expression on the membrane, we isolated membrane proteins using surface biotinylation followed by pull down of biotinylated protein with neutravidin beads and Western blotting (Figure 2D). Control pull down experiments in the absence of biotin demonstrate that both FL and Δ379 Panx1 channels as well as transferrin receptor (TfR) are present on the membrane surface while GAPDH and actin (as expected) are not (Figure S1). The multiple Panx1 bands on Western blots are due to the presence of multiple species with different levels of glycosylation [30]–[31]. Interestingly, the heavier complex-glycosylated form of Panx1 (known as gly2) appears to be absent in the Δ379 truncation mutant (Figure 2D). Furthermore the difference in size (between full-length Panx1 and Δ379) of the core non-glycosylated (gly0) form appears slightly larger than expected (10 kD vs. 5.2 kD) following truncation of the small 48-amino acid c-terminal peptide (out of 426 amino acids total), likely due to the fact that molecular weight and gel mobility are not always perfectly correlated.

It has been previously observed that full-length mouse Panx1 shows significant functional activity when expressed in HEK293T cells [7], [25], [32]. Since we did not observe this in our Yo-Pro influx assay, we performed patch clamp electrophysiology to examine this more closely. Following whole-cell voltage clamp, we set the holding voltage near the Panx1 reversal potential (−20 mV) and recorded carbenoxolone-sensitive currents within 10 min of achieving whole-cell voltage-clamp. All cells examined that were transfected with Δ379 (15 out of 15) had large (ave  =  approx. 1 nA at −100 mV) carbenoxolone-sensitive currents present (Figure 2E and Figures S3, S4) while mock transfected cells had very small carbenoxolone-sensitive currents (ave<100 pA at −100 mV). Some (2 out of 13) patched cells expressing the full-length mouse Panx1 channel (as identified by GFP co-transfection) showed significant currents that were much more outward-rectified compared to the Δ379 mutant (Figure S4). Such currents were more apparent after waiting >24 hours following transfection even though the FL mPanx1 channel is on the membrane surface by 15 hours. It is not clear whether this represents current passing through un-modified full-length Panx1 channels, or whether some full-length channels have been partially activated in some cells either through partial c-terminal cleavage, phosphorylation, or other processes that occur >24 hours following transient transfections. Such partial activation may be cell-type-dependent since it has been reported that FL mPanx1 currents from HEK293T cells appear smaller and more rectified than currents from SK-N-SH neuroblastoma cells [7]. To avoid the potential for such partial activation of Panx1 channels, we restricted our assays to 15–20 hours following transfection when full-length Panx1 was present on the membrane surface but inactive as measured by Yo-Pro influx. The Yo-Pro influx assay in particular provides a very large assay window between the inactive FL channel and active Δ379 mutant, allowing us to study the sequence determinants within the c-terminus that give rise to this functional difference.

**Figure 3:**
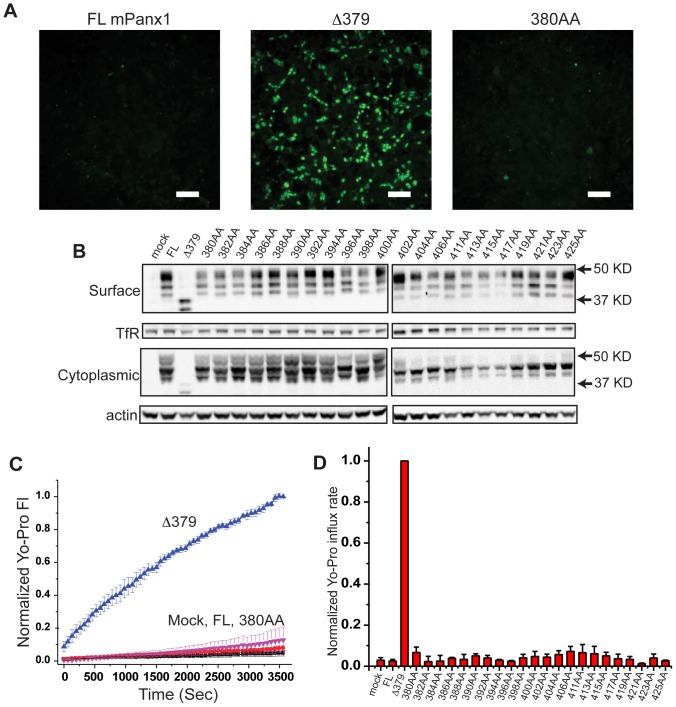
Panx1 channels with double-alanine substitutions within the c-terminus did not show detectable Yo-Pro-1 influx. (**A**) HEK293T cells were transfected with a series of double-alanine substitutions and assayed for Yo-Pro influx (example images shown). Scale bars are 100 µm. The Δ379 truncation mutant is used as a positive control for the assay. (**B**) All the substitution mutants were expressed on the membrane surface as shown by surface biotinylation-Western. (**C** and **D**) Pairwise Student's T-test comparisons show that there was no detectable increase in Yo-Pro influx over time (relative to mock transfected cells) for any of the double-alanine substitutions tested.

The c-terminal amino acids from 380 to 391 have been reported to play a role in modulation of human Panx1 as mutation of several key residues in this region resulted in a channel with measurable (though low) constitutive activity [25]. Since mouse Panx1 and human Panx1 are not well conserved in this region (Figure 1), we analyzed the role of c-terminal amino acids in mPanx1 by sequentially replacing pairs of residues with alanines from residues 380 (380AA) to 425 (425AA). Mutation of any single pair of residues in this region of mPanx1 did not result in a constitutively active channel as shown by lack of significant Yo-Pro influx over one hour for any of these constructs (Figure 3). This lack of activity was not the result of poor expression as evidenced by robust surface expression of all the double alanine mutant constructs as shown by Western blot of surface proteins (Figure 3B). Thus, in our hands, no single c-terminal residue is necessary for the ability of the c-terminus to keep the channel closed.

**Figure 4:**
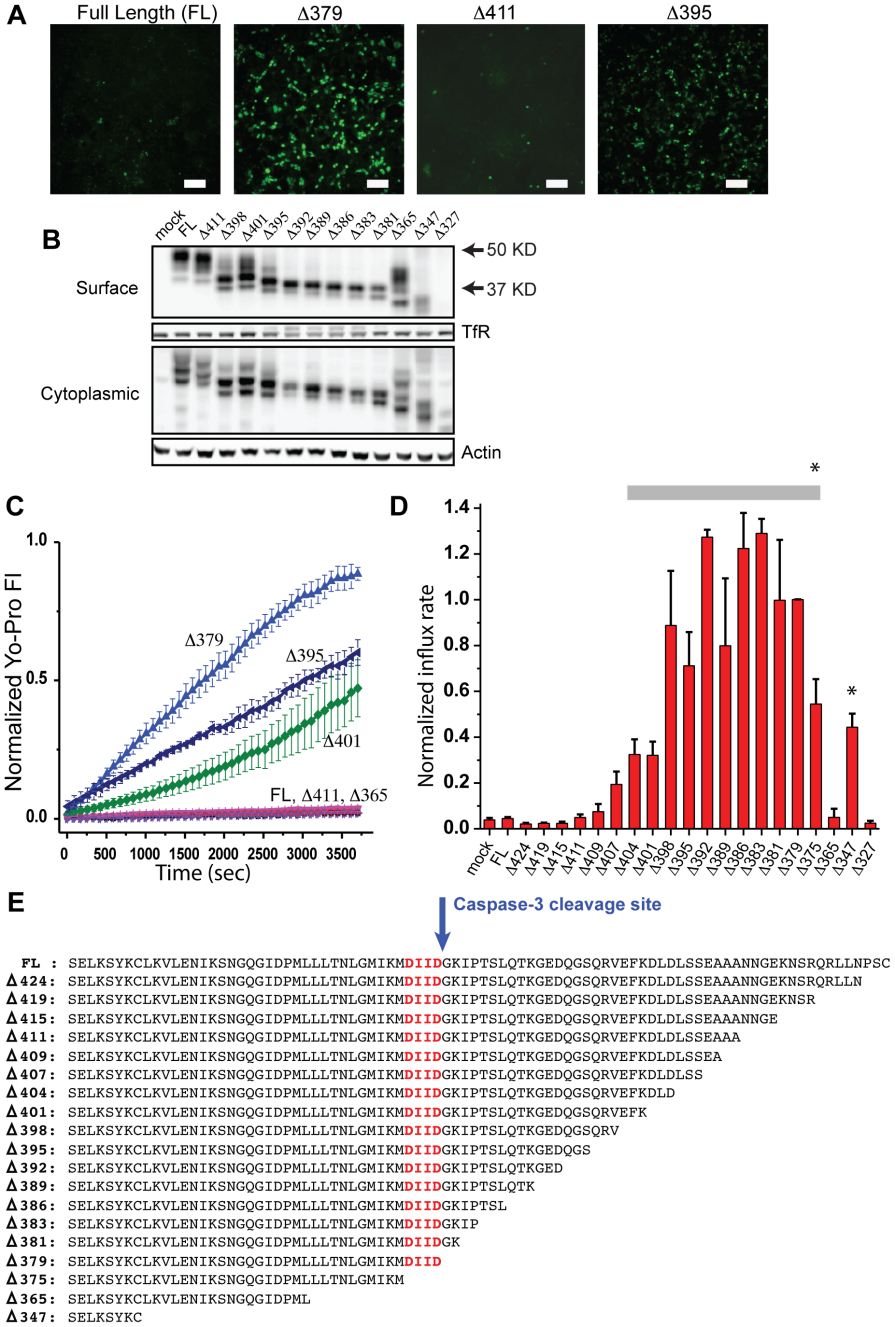
C-terminal truncations of mouse Panx1 shorter than Δ407 result in channels with detectable constitutive activity. (**A**) HEK293T cells were transfected with a series of Panx1 truncations and assayed for Yo-Pro influx with FL and Δ379 as negative and positive controls respectively (example images shown). Scale bars are 100 µm. (**B**) All truncations were expressed on the membrane surface except for Δ327 as shown by surface biotinylation-Western. (**C**) Yo-Pro influx was measured using fluorescence microscopy and (**D**) normalized slopes (normalized to the Δ379 control) of the initial 30 min were plotted. Statistical analysis was carried out for each individual experiment using a one-way ANOVA comparing the average Yo-Pro fluorescence between 3000 and 4000 sec for each mutant against the FL Panx1 control with Tukey-Kramer correction for multiple comparisons. The Yo-Pro fluorescence of each truncation mutant shorter than Δ407 and longer than Δ365 (as well as Δ347) was found to be significantly greater than that of FL control-transfected cells in all experiments (p<0.05). The length required for half-maximal Yo-Pro influx was determined to be 400.9 amino acids by fitting the data to a 3-parameter logistic function. (**E**) The c-terminal sequence of the truncated mutants are as shown.

We next investigated the role that the length of the c-terminus plays in the ability of Panx1 to allow constitutive Yo-Pro influx by making a set of truncation mutants with progressively smaller c-terminal lengths (Figure 4E). HEK293T cells transfected with truncated mutants showed detectable levels of Yo-Pro influx only with truncations shorter than Δ407. Individual experiments generally tested 4–6 mutants at one time (plus FL, and Δ379 controls) (see example in Figure 4C). Statistical analysis was carried out for each such experiment using a one-way ANOVA comparing the average Yo-Pro fluorescence between 3000 and 4000 sec for each mutant against the FL Panx1 control with Tukey-Kramer correction for multiple comparisons. The Yo-Pro fluorescence of each truncation mutant shorter than Δ407 and longer than Δ365 (as well as Δ347) was found to be significantly greater than that of FL control-transfected cells in all experiments (p<0.05) (as indicated in Figure 4D). The normalized rate of Yo-Pro influx over time was measured by calculating the slope of fluorescence increase during the first 30 minutes after addition of Yo-Pro, which increased as the c-terminal length was progressively shortened (Figure 4D).

All mutants with lengths equal to or greater than Δ407, were inactive (Figure 4). To test whether these truncation mutants were still expressed on the membrane surface, we measured surface expression by ICC (using two antibodies with distinct c-terminal epitopes – Figure S5) and Western blot of surface biotinylated proteins (Figure 4B and Table S1). All truncation mutants showed good surface expression with the exception of Δ327, which was not expressed. Δ365 was not active despite good surface expression. Interestingly, truncation mutants that showed significant Yo-Pro influx also showed absence of the gly2 species in Western blots of surface protein (Figure S7). The presence of the gly2 species was in general negatively correlated with Yo-Pro influx (Spearman's Rank Correlation Coefficient  = −0.871 indicating strong negative correlation).

**Figure 5:**
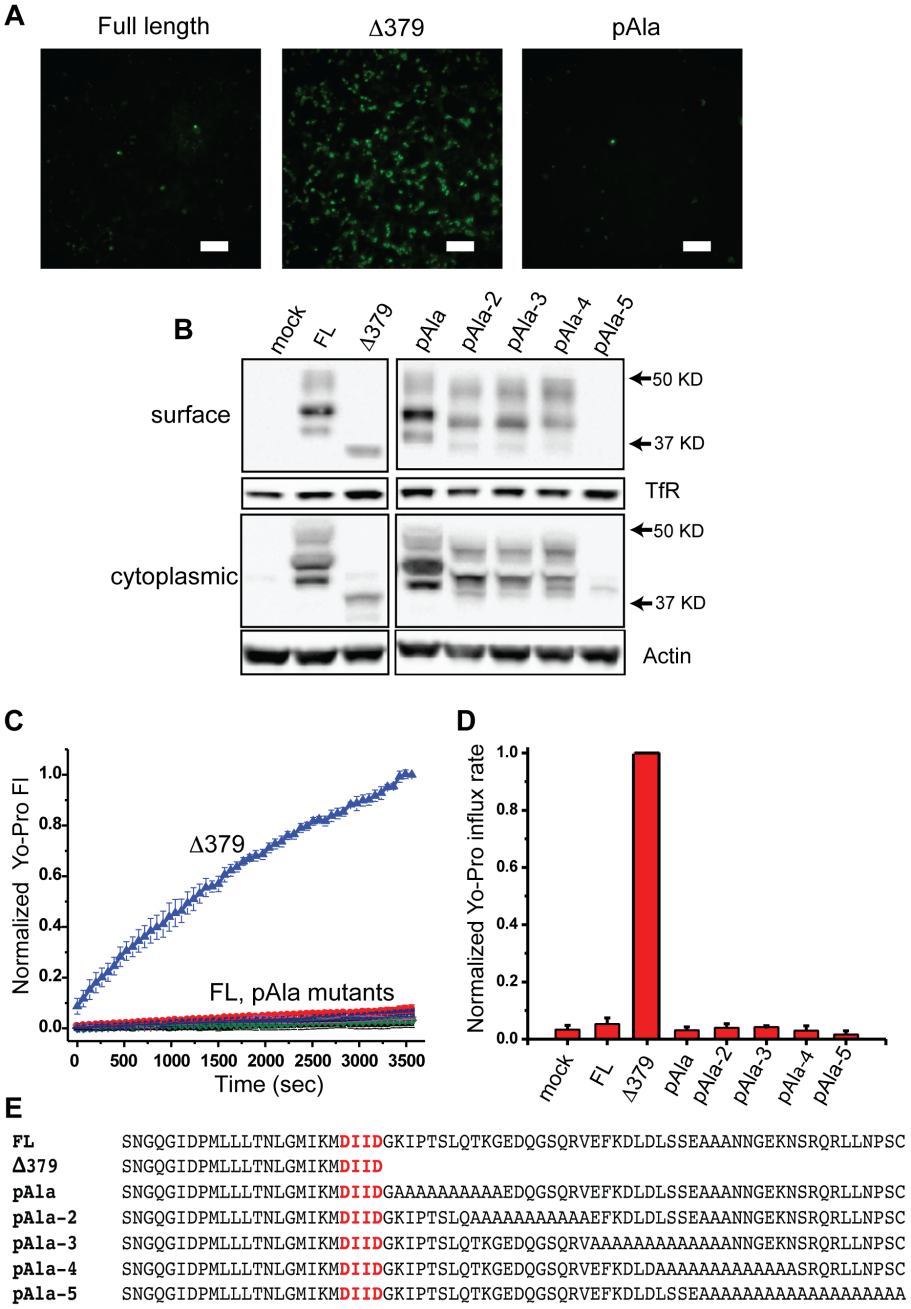
Poly-alanine (pAla) substitutions did not result in a constitutively active Panx1 channel. (**A**) HEK293T cells were transfected with a series of poly-alanine mutant Panx1 channels and assayed for Yo-Pro influx (example images shown). Scale bars are 100 µm. (**B**) All the poly-alanine mutants were expressed at the cell surface except for the outer tail-end substitution pAla-5, which did not express as determined by surface biotinylation-Western. (**C** and **D**) None of the poly-alanine mutants tested displayed any measurable Yo-Pro influx over time when compared with the Δ379 mutant. In particular, pairwise Student's T-test comparisons showed no statistically significant difference between mock, FL, and any of the poly-alanine mutants. (**E**) The c-terminal sequences of the poly-alanine mutants are as shown.

**Figure 6:**
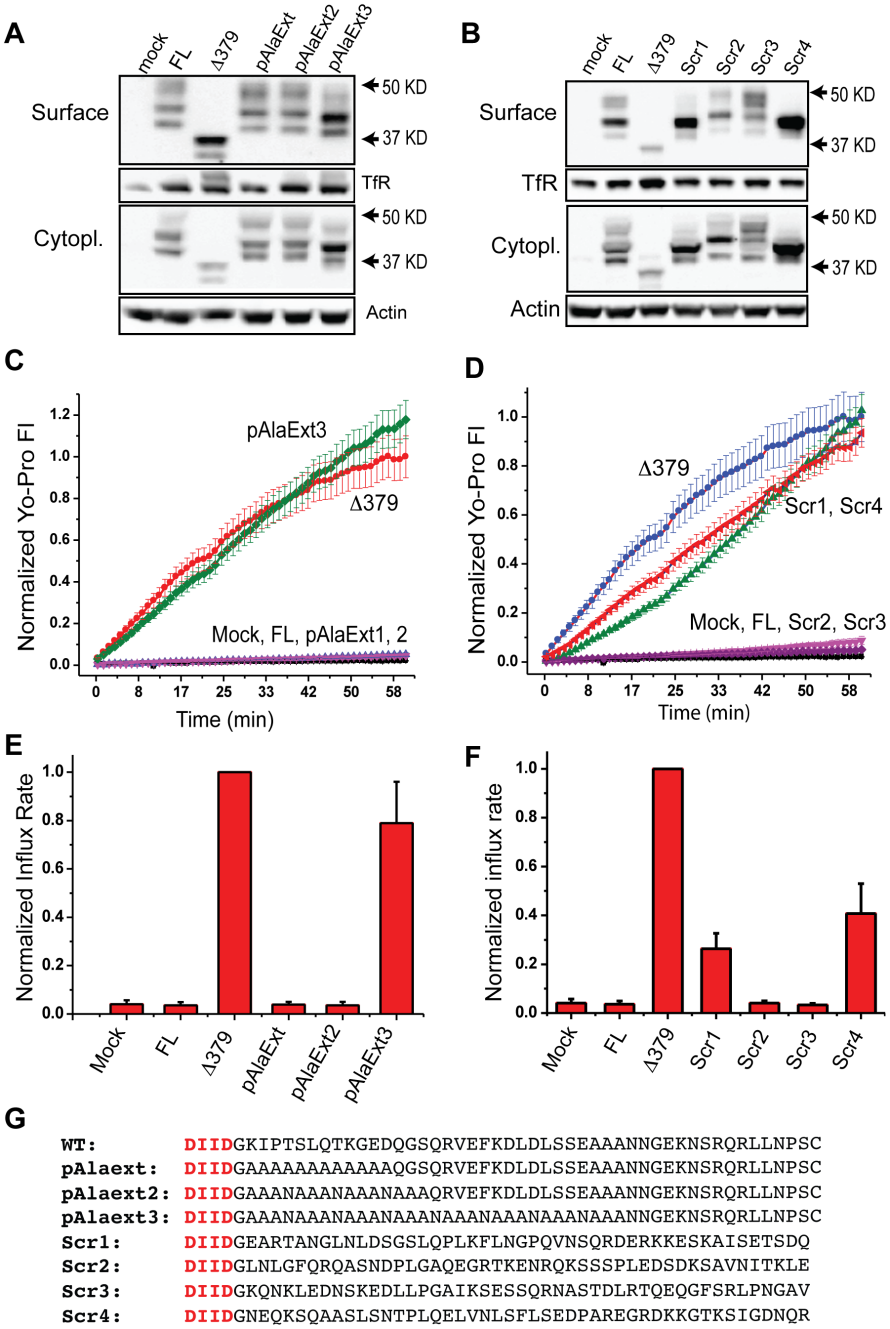
Activity of longer poly-alanine/asparagine Panx1 mutants as well as Panx1 mutants with scrambled c-termini. C-termini containing long poly-alanine repeats or completely scrambled c-termini were expressed and tested for Yo-Pro influx. As usual, FL and Δ379 served as negative and positive controls respectively. (**A** and **B**) All mutant channels were expressed on the cell surface as shown by surface biotinylation-Western. (**C** and **D**) Yo-Pro influx was measured using video microscopy and (**E** and **F**) normalized slopes of the initial 30 min were plotted. For each set of experiments, a one-way ANOVA with Tukey-Kramer multiple-comparison correction was used to show that pAlaExt, pAlaExt-2, Scr2, and Scr3 were not significantly different from mock or FL while pAlaExt-3 was constitutively active (p<0.05) and not different from Δ379. (**G**) Panx1 c-terminal sequences of tested mutants are as shown.

Since no single c-terminal amino acid residue is required to maintain Panx1 in the inactive state, we made more extreme mutations in an attempt to determine which part of the c-terminus is necessary to prevent Yo-Pro influx. Replacing stretches of residues with alanine repeats 10–12 residues in length failed to produce a channel allowing constitutive Yo-Pro influx (Figure 5). In particular, the pAla mutant (or pAlaExt in Figure 6) which replaces the entire region identified by Sandilos et al. [25] as a potential region required for interaction with the pore appears not to be necessary for maintaining mouse Panx1 in the inactive (closed) state. As before, we verified expression of all Panx1 mutants by ICC (Figure S6) and Western blot of surface proteins (Figure 5B), which demonstrates good expression on the membrane surface except for the pAla-5 mutant, which has no detectable membrane surface expression. To make sure this difference was not due to differences between mouse and human Panx1, we made the equivalent pAlaExt mutation in the human channel. This human pAlaExt mutant, like the mouse equivalent, showed no detectable Yo-Pro influx despite good surface expression (Figure S8), indicating that this region in human Panx1 is also not required to maintain the channel in the closed state.

Attempts to make longer alanine repeats resulted in technical issues due to the high GC content and repetitive nature of poly-alanine-encoding cDNAs. We therefore made constructs containing alanine repeats with asparagine residues placed every 4 residues to reduce the GC content at the DNA level (Figure 6G). Like the pAlaExt mutant, the pAlaExt-2 mutant containing three asparagine residues within a 15-residue polyalanine repeat resulted in a closed channel (Figure 6C, E). However, the longer pAlaExt-3 mutant allowed Yo-Pro influx similar to the Δ379 channel, indicating that the proximal 2/3rds of the c-terminus is necessary to maintain the channel in the closed state.

To further probe the importance of c-terminal amino acid residues in maintaining the channel in the closed state, we generated constructs with scrambled c-termini. These 4 constructs had c-termini of the same length and amino acid proportions but with scrambled order (Figure 6G). The sequence was scrambled from just after the caspase-cleavage site to the end of the FL Panx1 sequence. The c-terminal cysteine residue (which is not necessary to maintain the channel in the closed state) was removed so as to not cause spurious interactions via disulfide bonding. Scrambling itself was performed using the Shuffleseq program which is part of the European Molecular Biology Software Suite (EMBOSS) and is freely available online [28]. We find that some c-terminal scrambled constructs (src2 and src3) fail to show Yo-Pro influx (similar to mock and FL Panx1), while two scrambled constructs (scr1 and scr4) showed moderate Yo-Pro influx, indicating disruption of the ability of the c-terminus to keep the channel closed (Figure 6D, F). All of the scrambled constructs were expressed properly on the membrane surface as revealed by surface-Western (Figure 6B).

**Figure 7:**
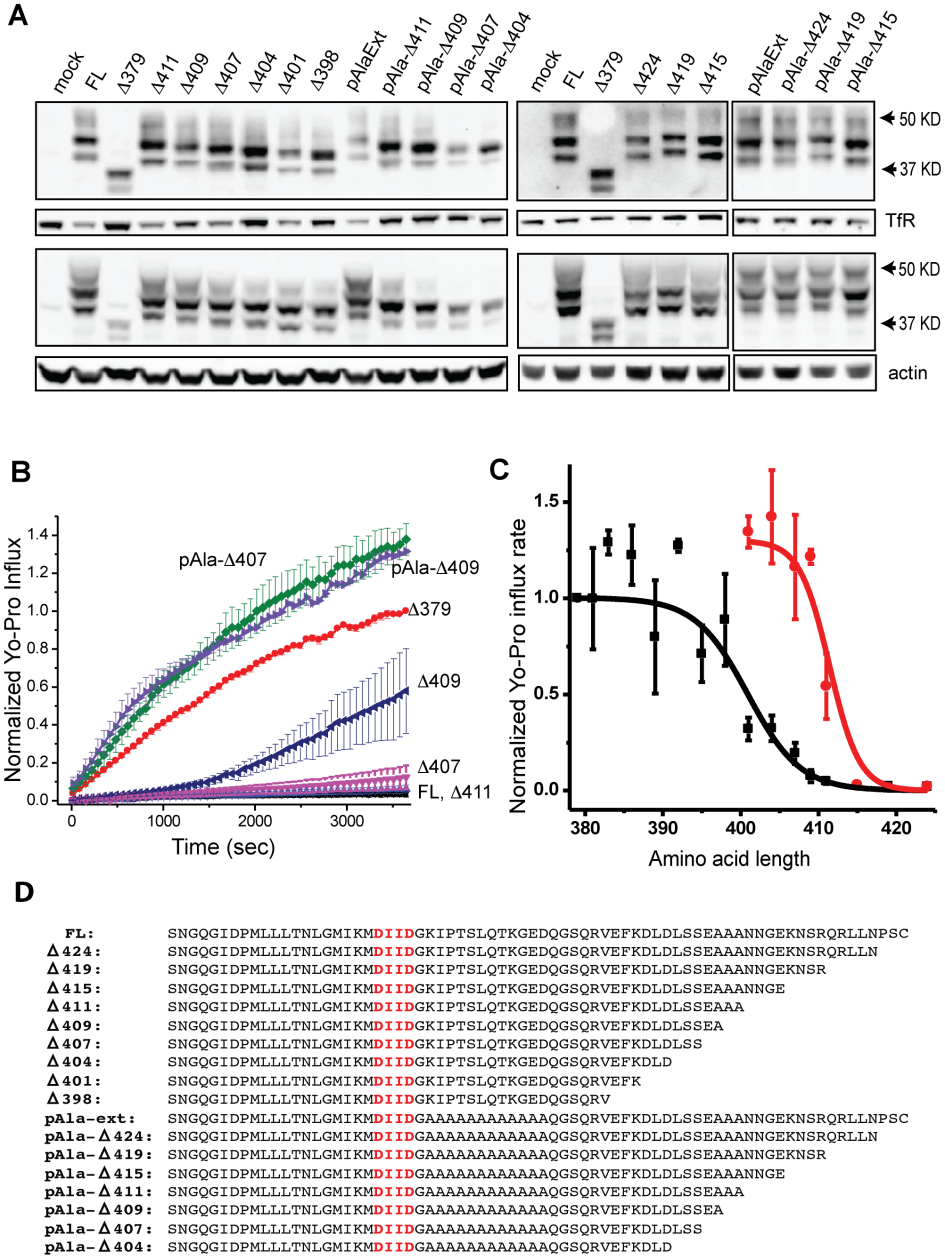
Presence of the pAlaExt mutation causes a 10-amino acid shift in the function-vs-length curve. C-terminal truncations of the pAlaExt mutation were tested for Yo-Pro influx side-by-side with truncations of the wild-type Panx1 channel. (**A**) All such mutant channels were expressed on the membrane surface as shown by surface biotinylation-Western. (**B**) The Yo-Pro influx of these mutants was measured using fluorescence microscopy and normalized slopes were calculated as before. (**C**) The presence of the pAlaExt mutation shifted the function-vs-length-dependence of c-terminal truncations by 10.5 amino acids, as determined by fitting both sets of data to a 3-parameter logistic function. The parameters were obtained as follows: for the wild-type channel, max  = 1.0, xc = 400.9, and slope factor  = −0.297. For the pAlaExt mutant channel, max  = 1.31, xc = 411.4, and slope factor(k) = −0.52. The logistic function used was y = max/(1+exp(−k*(x-xc))). (**D**) Sequences of the tested mutant Panx1 channel c-termini are as shown.

We find that the ability of the c-terminus to maintain the channel in the closed state depends on its length. Thus, truncation of the c-terminus up to Δ407 maintains this interaction and keeps the channel closed while further truncation results in constitutively open channels. We asked whether truncating the pAlaExt mutant would result in a different length dependence. Truncations of pAlaExt were made to the same lengths as the set of truncations of the FL Panx1 channel (see sequences in Figure 7D). Yo-Pro influx in cells transfected with the pAlaExt truncations was measured side-by-side with the corresponding FL Panx1 truncations (Figure 7C). As before, we verified membrane expression of all truncation mutants by surface-Western thus showing that all pAlaExt truncations were well expressed on the membrane surface (Figure 7A). Analysis of the initial slope of the Yo-Pro influx curves reveals that the length dependence of the pAlaExt truncations is shifted by approximately 10 residues toward the c-terminus (Figure 7B). Thus, while the pAlaExt mutation does not by itself result in a constitutively open channel, this mutation in conjunction with small truncations does result in constitutively open channels.

## Discussion

Our results indicate that while the 48 residue c-terminus (from the caspase-cleavage site to the c-terminal cysteine residue) is required to maintain Panx1 in the closed state, the interaction between the c-terminal peptide and the pore appears to be de-localized and non-specific. This conclusion is based on the fact that replacing each pair of c-terminal residues with alanines, or even large blocks of 10–15 residues with alanines/asparagines, fails to disrupt the ability of the c-terminus to maintain the channel in the closed state. Only replacing a stretch of 32 residues with alanines/asparagines (the pAlaExt-3 mutant) is able to fully disrupt this interaction. In fact, completely scrambling the c-terminal amino acid sequence was not sufficient in 2 out of 4 cases to disrupt this interaction. The interaction surface seems to involve residues in both the n-terminal and c-terminal sides of the 48-residue c-terminal peptide. Thus, while replacing the 12 amino acids immediately downstream of the caspase-cleavage site with alanines does not itself result in an open channel, coupling this modification with small c-terminal truncations does appear to force the channel into a constitutively open state. On the other hand, removal of the last 12 amino acids has no effect on the ability of the c-terminus to keep the channel closed even when the 12 amino acids immediately downstream of the caspase cleavage site are replaced with alanines, so perhaps these last 12 amino acids are not involved in the interaction at all (despite the fact that this region contains the most highly conserved part of the c-terminus).

As measured by biotinylation-pull down Western blots, the surface expression of the various Panx1 mutants are generally within a factor of 2-fold relative to the FL Panx1 channel with a few exceptions (see Table S1). In general, constitutively active mutants usually have lower surface expression than inactive mutants. To examine whether differences in surface expression between mutants might effect our conclusions, we re-analyzed our data correcting for measured relative surface expression levels (Fig. S9). We find that our conclusions remain unchanged with this analysis – i.e. none of the double-alanine or pAla mutants are constitutively active, pAlaExt3 is still active (while pAlaExt and pAlaExt2 are not), 2 of 4 scrambled mutants are inactive, truncation mutants shorter than Δ407 are active while longer truncations are active, and truncations in the context of the pAlaExt mutant have an activity vs. length curve shifted to the right by about 11 amino acids. The two scrambled mutants that are partially active (scr1 and scr4) appear less active when correcting for surface expression since these two mutants appear to have higher levels of surface expression relative to other mutants (see Table S1).

Such a large, non-specific and delocalized interaction surface contained within the 32 residues downstream of the caspase-cleavage site does not necessarily imply a low affinity interaction since many low affinity interactions within this large region could in theory add up to a high affinity interaction. However, a low affinity interaction may be tolerated in the case of Panx1 since the channel contains six identical subunits. This means that six 48-amino acid c-terminal gating peptides are positioned within interaction distance to the inner-vestibule of the pore. We calculate that the approximate concentration of c-termini within the inner vestibule region (approximating this region as a 100 Å cube) to be on the order of 10 mM. Therefore a low affinity interaction would still allow near 100% occupancy, especially if we assume that only a single c-terminus is required to block the pore at any one time. Thus, there is little evolutionary pressure to enhance the affinity or specificity of this interaction. This is likely the reason that this 48-residue c-terminus is very poorly conserved across different vertebrate species despite the fact that the c-terminus plays such a critical role in the gating and function of the Panx1 channel (see Figure 1). In fact there may be a selection pressure to maintain the low affinity nature of this interaction allowing the channel to be more sensitive to activation by caspase-3/7, as these enzymes would not need to remove all six c-termini to achieve channel opening.

While we can speculate that the interaction between pore and c-terminal gating peptide is likely very low affinity, measuring the actual affinity will be difficult. Experiments where the gating peptide is added exogenously will be complicated by the fact that very high concentrations likely are required to achieve the effective concentration normally present at the inner vestibule of the pore. Also, when presented exogenously, the gating peptide might bind to the pore/channel in ways normally not possible when the peptide is tethered, such as insertion into the pore in the opposite direction, making such affinity measurements inaccurate. A better way to measure the affinity of this interaction will be careful study of Panx1 channels engineered to have less than six gating peptides per hexamer. Modeling recordings of such channels at the single channel level should reveal the affinity of the gating peptide as well as the number of gating peptides required to block the pore.

Recently, it has been reported that single point mutations to alanine within the region immediately downstream of the caspase-cleavage site can cause partial opening of the human Panx1 channel [25], though this partial opening is only a small fraction of the full current observed in mutants truncated at the caspase cleavage site (and generally only observed at positive voltages). In our experiments, we did not observe any measurable Yo-Pro influx after alanine mutations within this region. In fact, we show that a mutant channel with this entire region replaced with a 12-amino acid alanine repeat (pAlaExt) is still expressed as a closed channel, even when the pAlaExt mutation is made in the context of the human Panx1 channel. One difference is that we have used Yo-Pro influx as our readout vs. whole cell patch clamp recordings, although the pAlaExt mutant also shows very little current by whole-cell patch clamp in our hands (see Figures S3–S4). Using Yo-Pro influx as our readout gives us the advantage of being able to measure activity from many cells simultaneously as well as allowing simultaneous measurements of multiple mutant channels in one experiment. The higher-throughput nature of the Yo-Pro influx technique in particular allows us to more easily restrict our analysis to time-points <20 hours following transfection to avoid potential interference due to cell-stress-induced Panx1 activation and also provides us with a much larger assay window. One potential disadvantage of using Yo-pro influx for our assays relative to whole cell patch clamp is that we might miss mutants that allow constitutive flux of small ions but not large molecules such as Yo-pro. While this is a possibility, we have not observed this with any of the mutants we have looked at by patch clamp.

During the course of this study, we observed that constitutively-active mutant Panx1 channels show reduced levels of the gly2 form of the channel. This was not expected since mutations within the c-terminus are distant from the extracellular N254 glycosylation site. We speculate that constitutively active Panx1 channels disrupt many intracellular processes (such as glycosylation) because of the fact that ATP, amino acids, and other small molecules are being drained from the cytoplasm as a result of permeation through constitutively open Panx1 channels. We also observe reduced expression of Panx1 itself and GFP/dsRed when cells are transfected with an active Panx1 mutant, possibly as a result of the same small-molecule depletion process.

Here we provide evidence that the c-terminal gating peptide maintains the channel in the closed state though a large delocalized and non-specific interaction surface. The most likely gating mechanism is direct plugging of the pore by the c-terminal peptide interacting within the pore via multiple low-affinity interactions. Such a gating mechanism is similar to ball-and-chain gating that occurs in the case of N-type inactivation in voltage-gated K^+^ channels and fast inactivation in voltage-gated Na^+^ channels [33]–[34], though in these cases the interaction surfaces are smaller and more specific, especially in the case of Na^+^ channels where rather subtle single-amino acid modifications within a hydrophobic patch in the intracellular loop between domains III and IV can completely eliminate the interaction [34]. The ball-and-chain interaction in Na^+^ channels likely needs to have reasonably high-affinity since only one such gating peptide exists per channel and the interaction needs to occur within 1–2 msec, unlike in the case of Panx1 where 6 identical gating peptides exist near a single pore and a much slower interaction time-course is probably acceptable. Interestingly, it has been shown that Panx1 can be activated in a reversible manner without cleavage of the c-terminus [10], [14], [21]. Future experiments will be required to understand why the intact c-terminus is unable to keep the channel closed under these conditions. Understanding how Panx1 becomes active under various physiological or pathophysiological conditions will help to better define how Panx1 activity is involved in these processes.

## Supporting Information

Figure S1 **Full-length mPanx1 and select Panx1 mutants are membrane-expressed.** HEK293T cells expressing either FL mPanx1 or various Panx1 mutants (Δ379, pAlaExt, Δ409, pAla-Δ409) were subjected to the surface biotinylation reaction with or without added biotin. Lysates were then processed identically using neutravidin beads to isolate biotinylated proteins and run on SDS-PAGE gels. Western blots were prepared from the gels and probed for either Panx1 (mid antibody), actin, transferrin receptor (TfR), or GAPDH. The cytoplasmic fractions were also run side-by-side. Panx1 and TfR were found in the surface fraction when biotin was added but not when biotin was left out of the reaction. Small amounts of actin and GAPDH were also present in the surface fraction, but their presence did not depend on biotin and thus represent non-specific adsorption to the neutravidin beads. Thus, Panx1, Panx1 mutants, and TfR were found to be surface exposed while actin and GAPDH were not, as expected.
(TIF)

Figure S2 **Biotinylation of surface Panx1 and Panx1 mutants is not affected by Panx1 channel expression.** Cells expressing either full length mPanx1 or two Panx1 mutants (Δ379, pAlaExt) were subjected to surface biotinylation in the presence or absence of 50 µM carbenoxolone (which is able to fully block Yo-Pro influx and ionic current through open Panx1 channels). Lysates were processed to isolate biotinylated proteins as well as cytoplasmic proteins and run on an SDS-PAGE gel. The resulting Western blot was probed for mPanx1, TfR, and actin. No difference in the amount of Panx1, Panx1 mutants, or TfR protein was observed with and without carbenoxolone added to the biotinylation reaction under our experimental conditions.
(TIF)

Figure S3 **Example recordings of HEK293T cells expressing mPanx1 or Panx1 mutants.** Currents from HEK293T cells expressing either pCDNA3.1 vector-only (mock), FL mPanx1 or Panx1 mutants (Δ379, pAlaExt) were recorded using whole cell patch clamp. (**A**) Current-voltage (I-V) curves were obtained by holding the membrane voltage at −20 mV (near the reversal potential) and applying a ramp voltage protocol from −100 mV to +60 mV in the absence and presence of 100 µM carbenoxolone (example shown is from a Δ379-expressing cell). (**B**) Between 8–15 cells of each group were patched and carbenoxolone-sensitive currents of 3 typical cells of each are shown here. Cells transfected with Δ379 show large currents, FL- and pAlaExt-transfected cells show much smaller currents (only at positive voltages), and vector-only transfected cells show extremely small currents.
(TIF)

Figure S4 **Carbenoxolone-sensitive currents recorded from cells transfected with full-length or mutant Panx1 channels.** HEK293T cells were transfected with FL Panx1, Δ379, pAlaExt, or pCDNA3.1 vector alone (mock). (**A**) Average carbenoxolone-sensitive currents (n = 8–15 cells) recorded at −100 mV were much larger in Δ379-expressing cells relative to FL or pAlaExt cells. (**B**) Similarly, average carbenoxolone-sensitive currents (n = 8–15 cells) recorded at +60 mV were much larger in Δ379-expressing cells relative to FL or pAlaExt cells. A one-way ANOVA comparing all pairs of mean slopes with Tukey-Kramer correction for multiple comparisons was used to show that Δ379 has significantly higher current at both −100 mV and +60 mV compared to mock, FL, or pAlaExt (p<0.05), while FL and pAlaExt were not significantly different than mock (though there was a trend toward significance in the case of FL at +60 mV). (**C**) In particular, we found that some (2 out of 13) cells expressing FL mPanx1 had detectable carbenoxolone-sensitive currents that appeared to be much more outward-rectified relative to currents seen in cells expressing Δ379.
(TIF)

Figure S5 **Staining of cells expressing Panx1 truncation mutants by immunocytochemistry.** (**A**) Two different polyclonal antibodies with distinct c-terminal epitopes (**B**) were used for staining fixed/permeabilized HEK293T cells expressing either FL Panx1 or one of the various inactive truncation mutants (Δ424, Δ419, Δ415, Δ411, Δ409, Δ407, Δ404). YZ2868 was only able to stain cells expressing FL Panx1 or Δ424 as further truncation appears to disrupt the epitope. Staining with YZ2865 however showed expression on the membrane of all of these constructs. In the case of each of these mutants, staining appeared consistent with membrane expression. Scale bars for images are 10 µm. (**C**) The c-terminal sequences of the truncation mutants as well as the locations of the peptides used to generate the antibodies are shown.
(TIF)

Figure S6 **Staining of cells expressing Panx1 poly-alanine mutants by immunocytochemistry.** (**A**) HEK293T cells expressing one of the various inactive pAla mutants (pAla, pAla-2, pAla-3, pAla-4, pAlaExt, pAlaExt-2) were stained with two polyclonal antibodies with distinct c-terminal epitopes, YZ2865 and YZ2686. Both antibodies were able to stain all of the pAla mutants with the exception of pAla-2, which showed no staining with YZ2865 likely due to disruption of the epitope. In the case of each of these mutants, staining appeared consistent with membrane expression. Scale bars for images are 10 µm. (**B**) The sequences of the mutants tested as well as the location of the peptides used to generate the antibodies are shown.
(TIF)

Figure S7 **The absence of the heavily-glycosylated form of Panx1 (gly2) correlates with constitutive functional activity.** To quantify the amount of gly2 form present when the various Panx1 mutants were expressed in this study, we measured the density of the gly0, gly1, and gly2 bands from Western blots of the surface fractions. (**A**) We calculated the ratio gly2: (gly0+gly1) for each mutant and averaged together data from 2–3 Western blots per mutant. Error bars represent SEMs calculated for each mutant. (**A-below**) Plotted along side are the normalized Yo-Pro influx rates measured for each of these mutants. In general, mutants with high Yo-Pro influx rates have low levels of gly2, whereas mutants with low Yo-Pro influx rates have high levels of gly2 relative to gly0 and gly1. (**B**) To further examine this correlation, we plotted the log of the normalized Yo-Pro influx rate against the log of the gly2:(gly0+gly1) ratio. The line shown in grey shows the overall trend in the data but in no way implies that this relationship should be linear. To determine whether this apparent correlation is significant, we calculated the Spearman's Rank Correlation Coefficient, which determines whether a correlation exists in a non-parametric way without any assumption as to the functional form of this correlation. In this case, we find that the Spearman's Rank Correlation Coefficient  = −0.871 indicating strong negative correlation.
(TIF)

Figure S8 **Human Panx1 (Δ379), but not human full-length or pAlaExt, is constitutively active.** Human FL Panx1 as well as human mutants Δ379 and pAlaExt and pCDNA3.1 vector-only (mock) were transiently expressed in HEK293T cells. (**A**) Surface biotinylation- Western blots show that the FL human Panx1 channel as well as the human Δ379 and pAlaExt mutants are expressed in the membrane surface at very similar levels to the equivalent mPanx1 constructs. (**B**) As in the case of the equivalent mouse Panx1 channel, human Δ379 showed robust Yo-Pro influx following addition of 1 µg/mL Yo-Pro-1 while human FL and pAlaExt showed no detectable Yo-Pro influx after 50 min, similar to vector-only transfected cells.
(TIF)

Figure S9 **Re-analysis of Yo-Pro influx data while correcting for surface expression differences.** The Yo-Pro influx rate data collected for each Panx1 mutant was corrected to account for differences in surface expression level of each mutant. Briefly, we divided Yo-Pro influx rate by relative surface expression level to obtain a corrected Yo-Pro influx rate. The new error bars represent SEM corrected to account for propagation of error. Thus, while the data have been re-normalized to the Δ379 mutant, the Δ379 mutant now has a measurable SEM resulting from the error in the measurement of expression level. Shown here are (**A**) corrected and re-normalized Yo-Pro influx rate for the double-alanine mutants, (**B**) the pAla mutants, (**C**) the pAlaExt mutants, (**D**) the scrambled mutants, and (**E**) the truncation mutants. (**F**) The presence of the pAlaExt mutation shifted the expression-corrected function-vs-length-dependence of c-terminal truncations by 11.5 amino acids, as determined by fitting both sets of truncation mutant data to a 3-parameter logistic function. The parameters were obtained as follows: for the wild-type channel, max  = 1.0, xc = 397.2, and slope factor  = −0.293. For the pAlaExt mutant channel, max  = 1.25, xc = 408.6, and slope factor(k) = −0.72. The logistic function used was y = max/(1+exp(−k*(x-xc))).
(TIF)

Table S1 **Surface expression and constitutive activity of Panx1 mutants examined in this study.** Membrane surface expression (as determined by analysis of biotinylation-pull down Western blots) and Yo-Pro influx rate for each mutant Panx1 channel are shown. Results are presented as mean ± standard error of the mean (SEM).
(DOC)
