# Supplementary figures and images for: Pannexin-1 Is Blocked by Its C-Terminus through a Delocalized Non-Specific Interaction Surface
Source: PLoS One. 2014 Jun 9;9(6):e99596. doi: 10.1371/journal.pone.0099596 (PMC4049774; doi:10.1371/journal.pone.0099596)

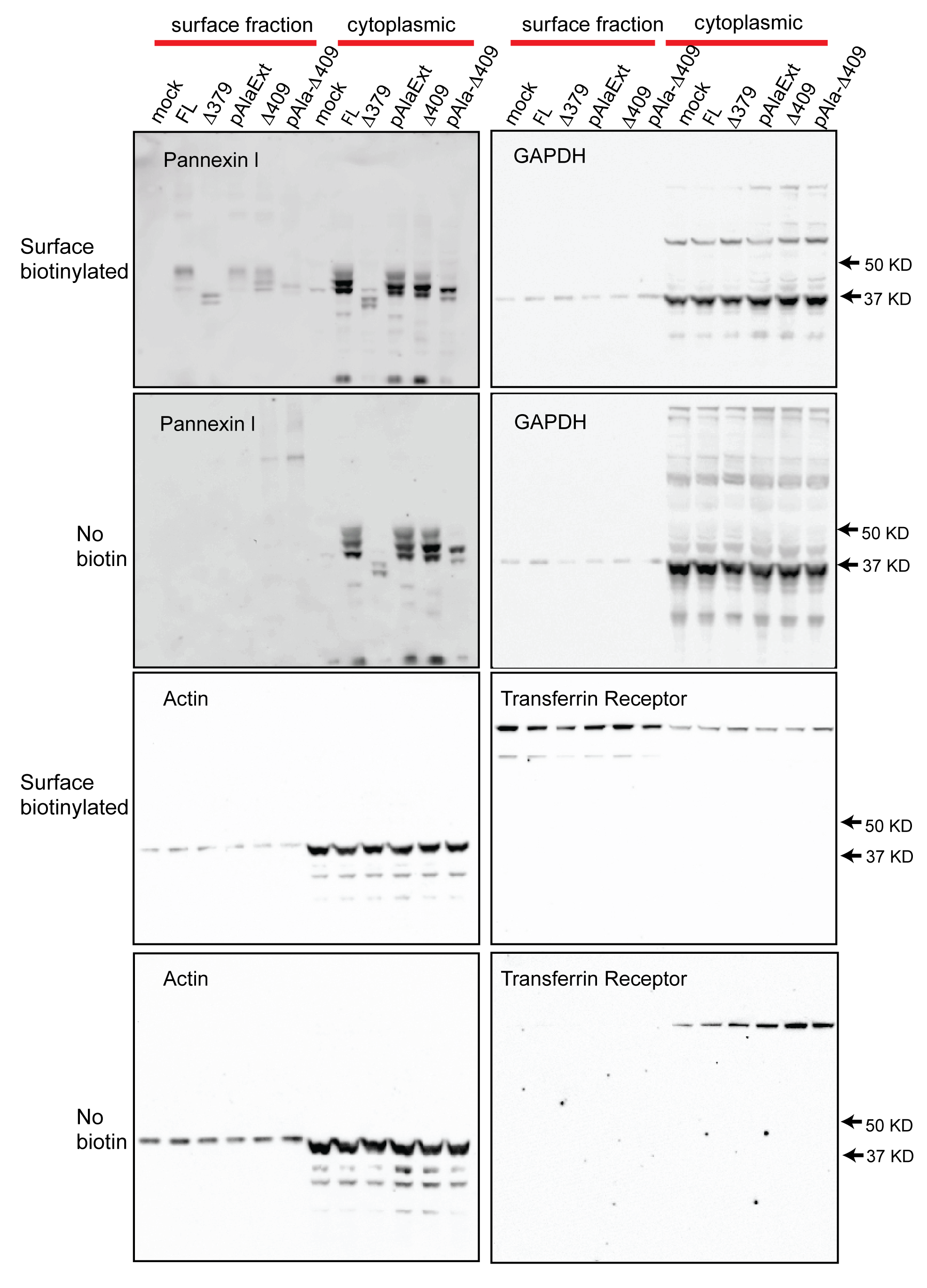

Supplement: Figure S1 — Full-length mPanx1 and select Panx1 mutants are membrane-expressed. HEK293T cells expressing either FL mPanx1 or various Panx1 mutants (Δ379, pAlaExt, Δ409, pAla-Δ409) were subjected to the surface biotinylation reaction with or without added biotin. Lysates were then processed identically using neutravidin beads to isolate biotinylated proteins and run on SDS-PAGE gels. Western blots were prepared from the gels and probed for either Panx1 (mid antibody), actin, transferrin receptor (TfR), or GAPDH. The cytoplasmic fractions were also run side-by-side. Panx1 and TfR were found in the surface fraction when biotin was added but not when biotin was left out of the reaction. Small amounts of actin and GAPDH were also present in the surface fraction, but their presence did not depend on biotin and thus represent non-specific adsorption to the neutravidin beads. Thus, Panx1, Panx1 mutants, and TfR were found to be surface exposed while actin and GAPDH were not, as expected. (TIF) [file pone.0099596.s001.tif]

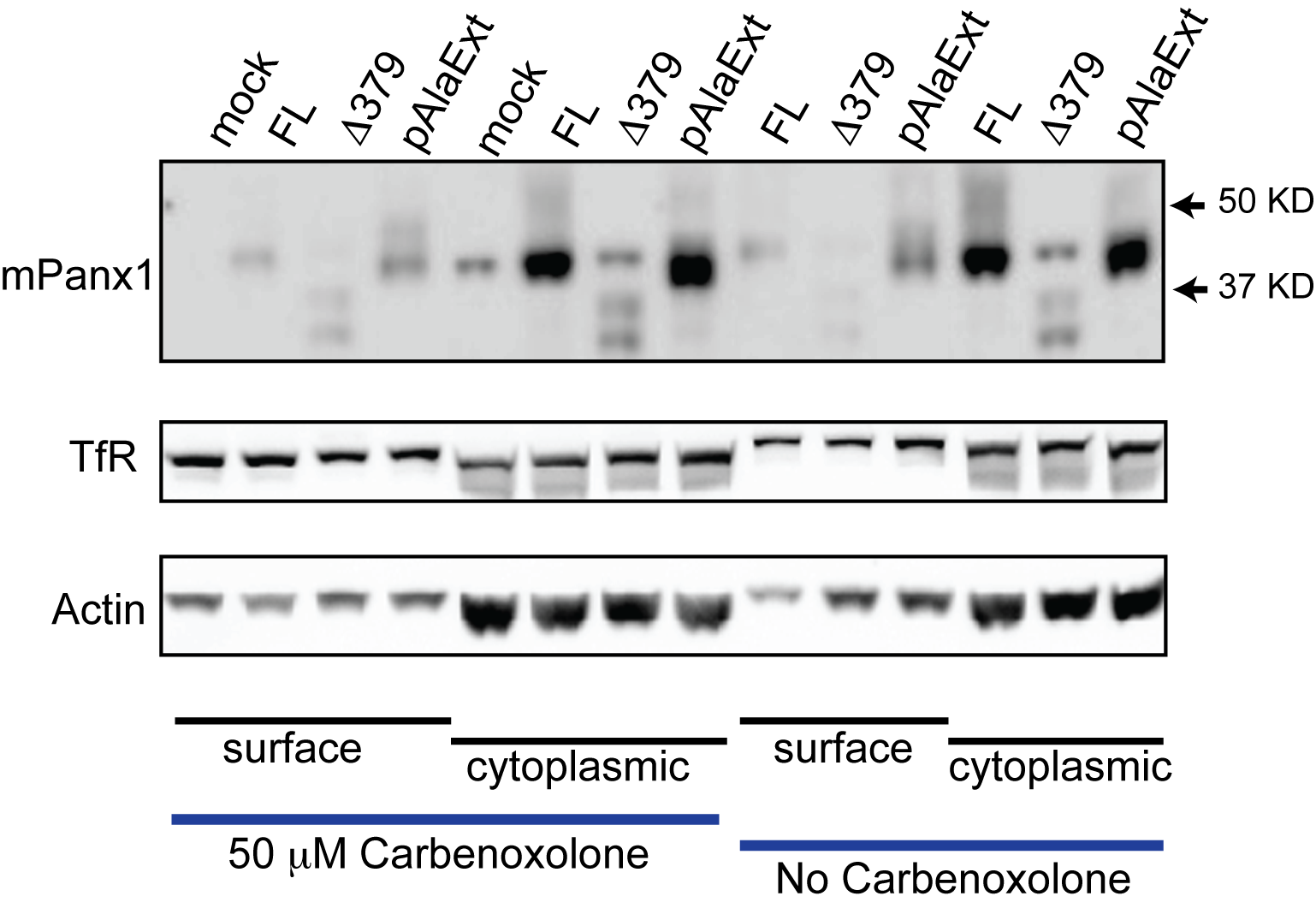

Supplement: Figure S2 — Biotinylation of surface Panx1 and Panx1 mutants is not affected by Panx1 channel expression. Cells expressing either full length mPanx1 or two Panx1 mutants (Δ379, pAlaExt) were subjected to surface biotinylation in the presence or absence of 50 µM carbenoxolone (which is able to fully block Yo-Pro influx and ionic current through open Panx1 channels). Lysates were processed to isolate biotinylated proteins as well as cytoplasmic proteins and run on an SDS-PAGE gel. The resulting Western blot was probed for mPanx1, TfR, and actin. No difference in the amount of Panx1, Panx1 mutants, or TfR protein was observed with and without carbenoxolone added to the biotinylation reaction under our experimental conditions. (TIF) [file pone.0099596.s002.tif]

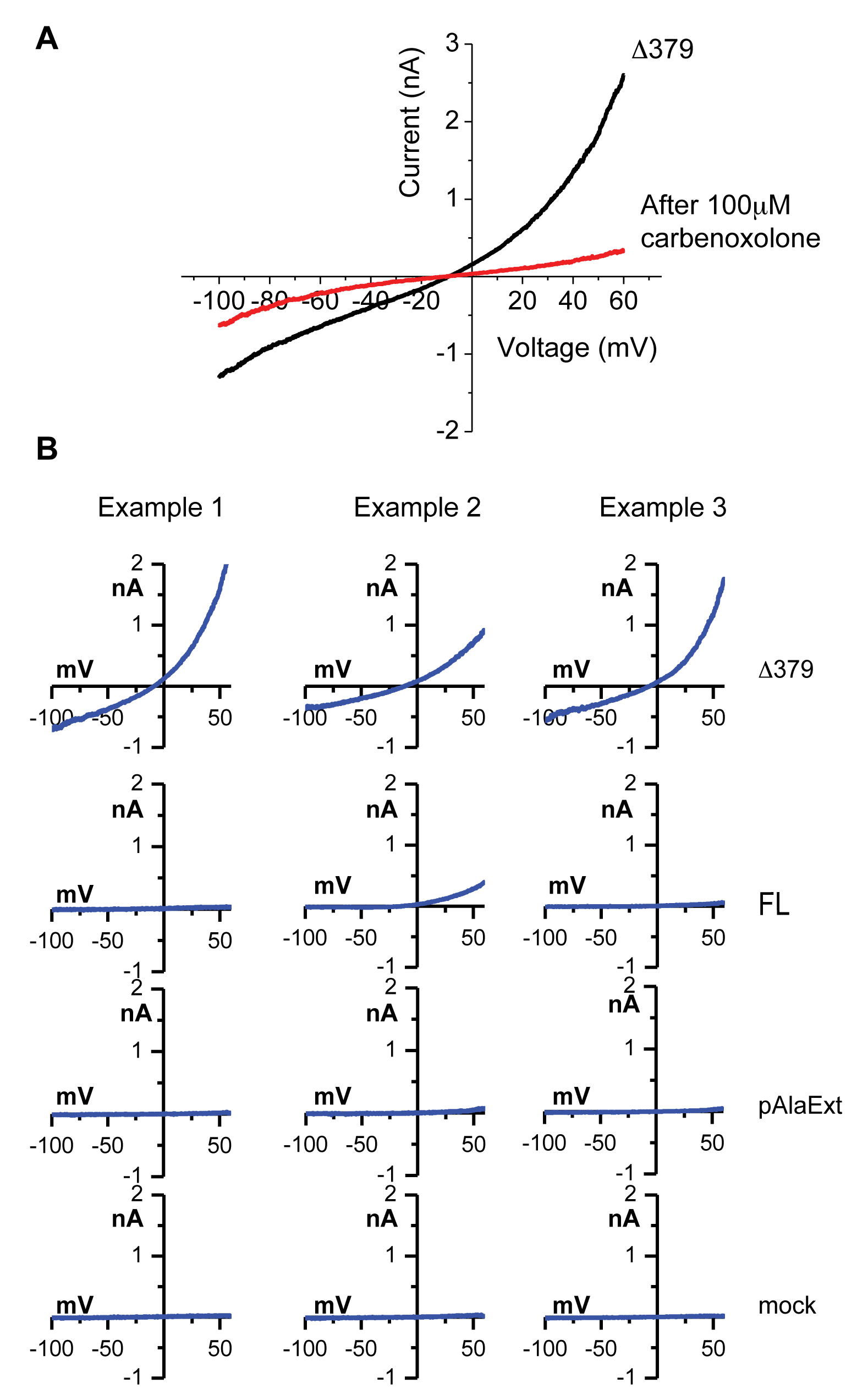

Supplement: Figure S3 — Example recordings of HEK293T cells expressing mPanx1 or Panx1 mutants. Currents from HEK293T cells expressing either pCDNA3.1 vector-only (mock), FL mPanx1 or Panx1 mutants (Δ379, pAlaExt) were recorded using whole cell patch clamp. (A) Current-voltage (I-V) curves were obtained by holding the membrane voltage at −20 mV (near the reversal potential) and applying a ramp voltage protocol from −100 mV to +60 mV in the absence and presence of 100 µM carbenoxolone (example shown is from a Δ379-expressing cell). (B) Between 8–15 cells of each group were patched and carbenoxolone-sensitive currents of 3 typical cells of each are shown here. Cells transfected with Δ379 show large currents, FL- and pAlaExt-transfected cells show much smaller currents (only at positive voltages), and vector-only transfected cells show extremely small currents. (TIF) [file pone.0099596.s003.tif]

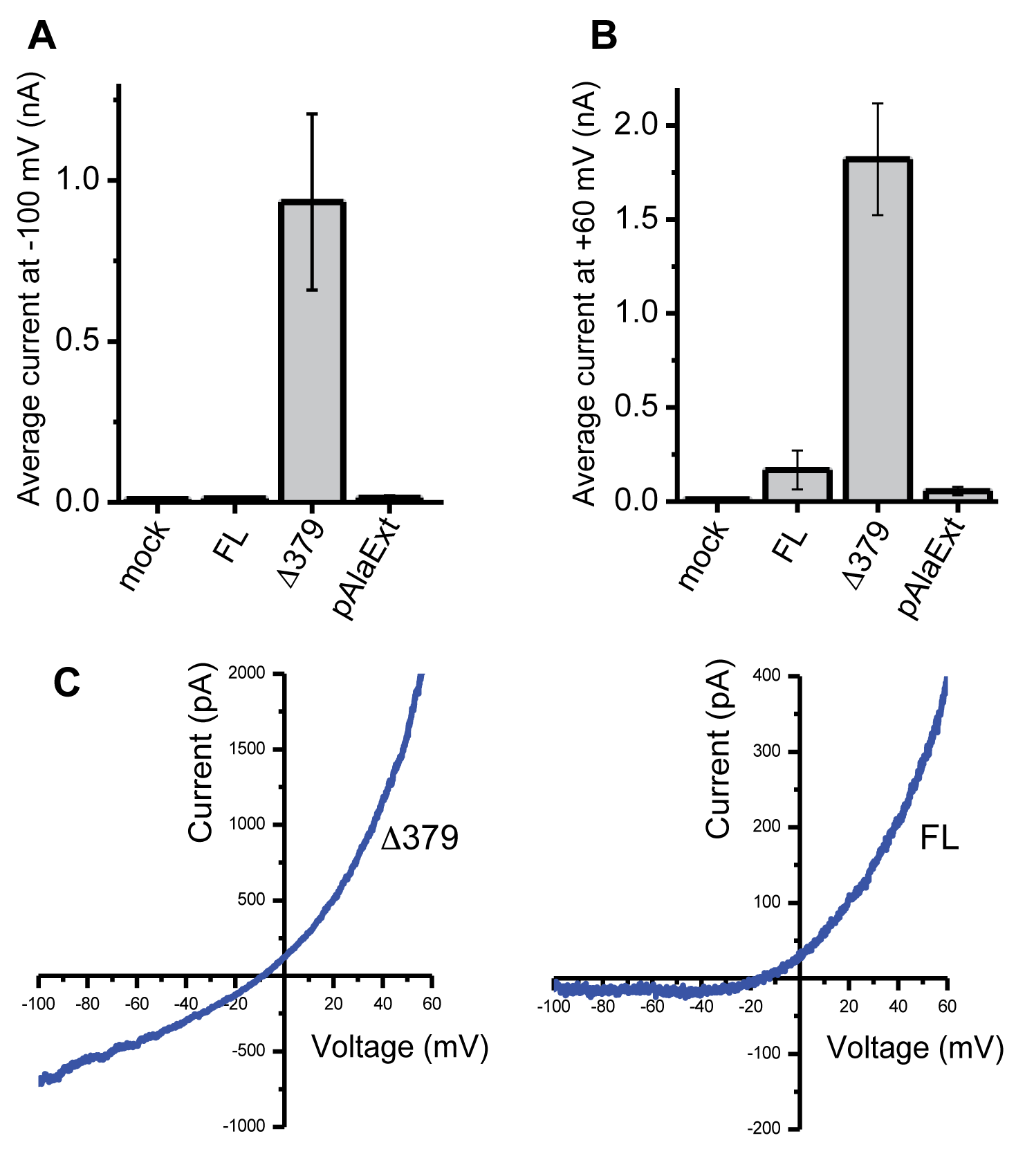

Supplement: Figure S4 — Carbenoxolone-sensitive currents recorded from cells transfected with full-length or mutant Panx1 channels. HEK293T cells were transfected with FL Panx1, Δ379, pAlaExt, or pCDNA3.1 vector alone (mock). (A) Average carbenoxolone-sensitive currents (n = 8–15 cells) recorded at −100 mV were much larger in Δ379-expressing cells relative to FL or pAlaExt cells. (B) Similarly, average carbenoxolone-sensitive currents (n = 8–15 cells) recorded at +60 mV were much larger in Δ379-expressing cells relative to FL or pAlaExt cells. A one-way ANOVA comparing all pairs of mean slopes with Tukey-Kramer correction for multiple comparisons was used to show that Δ379 has significantly higher current at both −100 mV and +60 mV compared to mock, FL, or pAlaExt (p<0.05), while FL and pAlaExt were not significantly different than mock (though there was a trend toward significance in the case of FL at +60 mV). (C) In particular, we found that some (2 out of 13) cells expressing FL mPanx1 had detectable carbenoxolone-sensitive currents that appeared to be much more outward-rectified relative to currents seen in cells expressing Δ379. (TIF) [file pone.0099596.s004.tif]

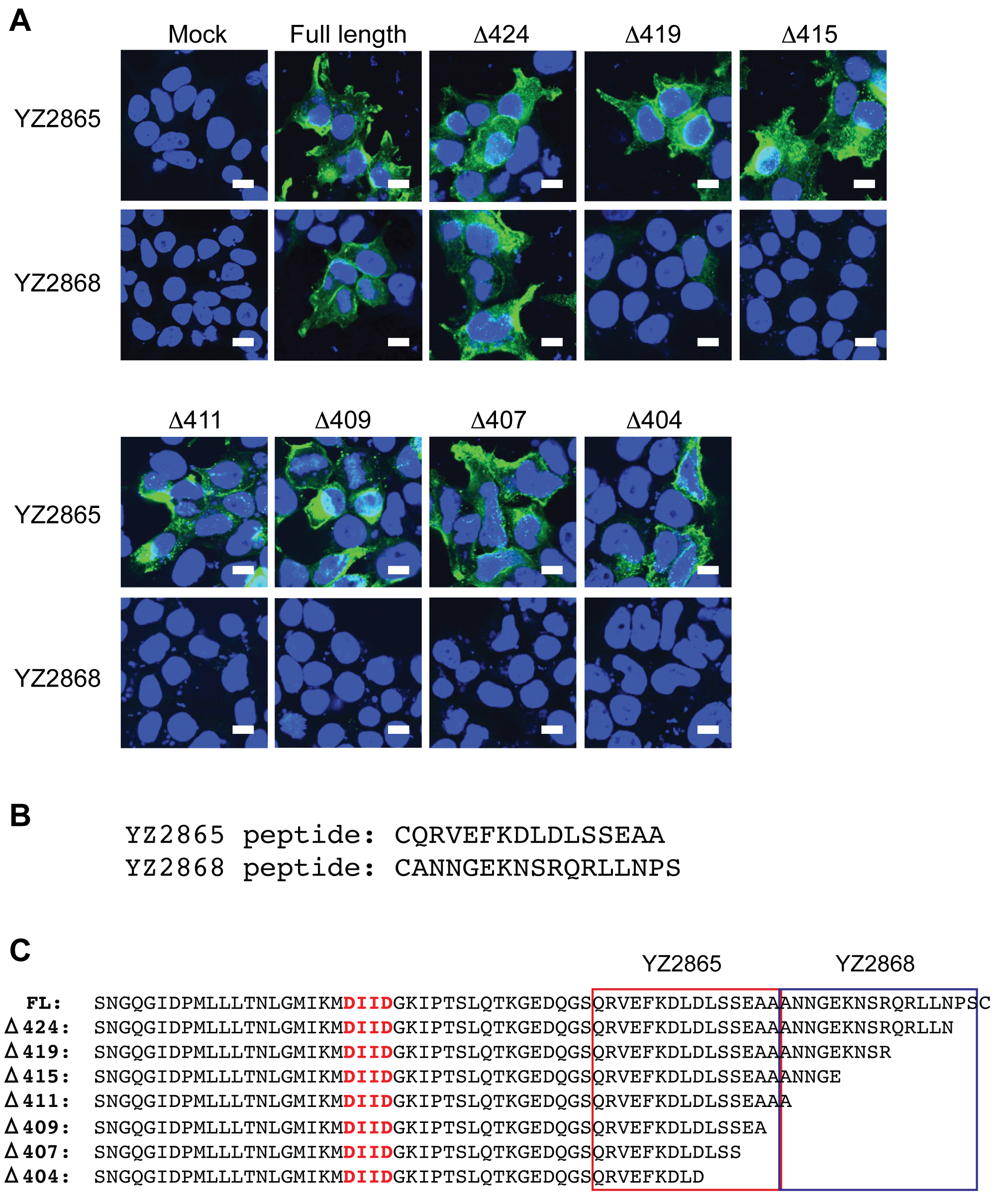

Supplement: Figure S5 — Staining of cells expressing Panx1 truncation mutants by immunocytochemistry. (A) Two different polyclonal antibodies with distinct c-terminal epitopes (B) were used for staining fixed/permeabilized HEK293T cells expressing either FL Panx1 or one of the various inactive truncation mutants (Δ424, Δ419, Δ415, Δ411, Δ409, Δ407, Δ404). YZ2868 was only able to stain cells expressing FL Panx1 or Δ424 as further truncation appears to disrupt the epitope. Staining with YZ2865 however showed expression on the membrane of all of these constructs. In the case of each of these mutants, staining appeared consistent with membrane expression. Scale bars for images are 10 µm. (C) The c-terminal sequences of the truncation mutants as well as the locations of the peptides used to generate the antibodies are shown. (TIF) [file pone.0099596.s005.tif]

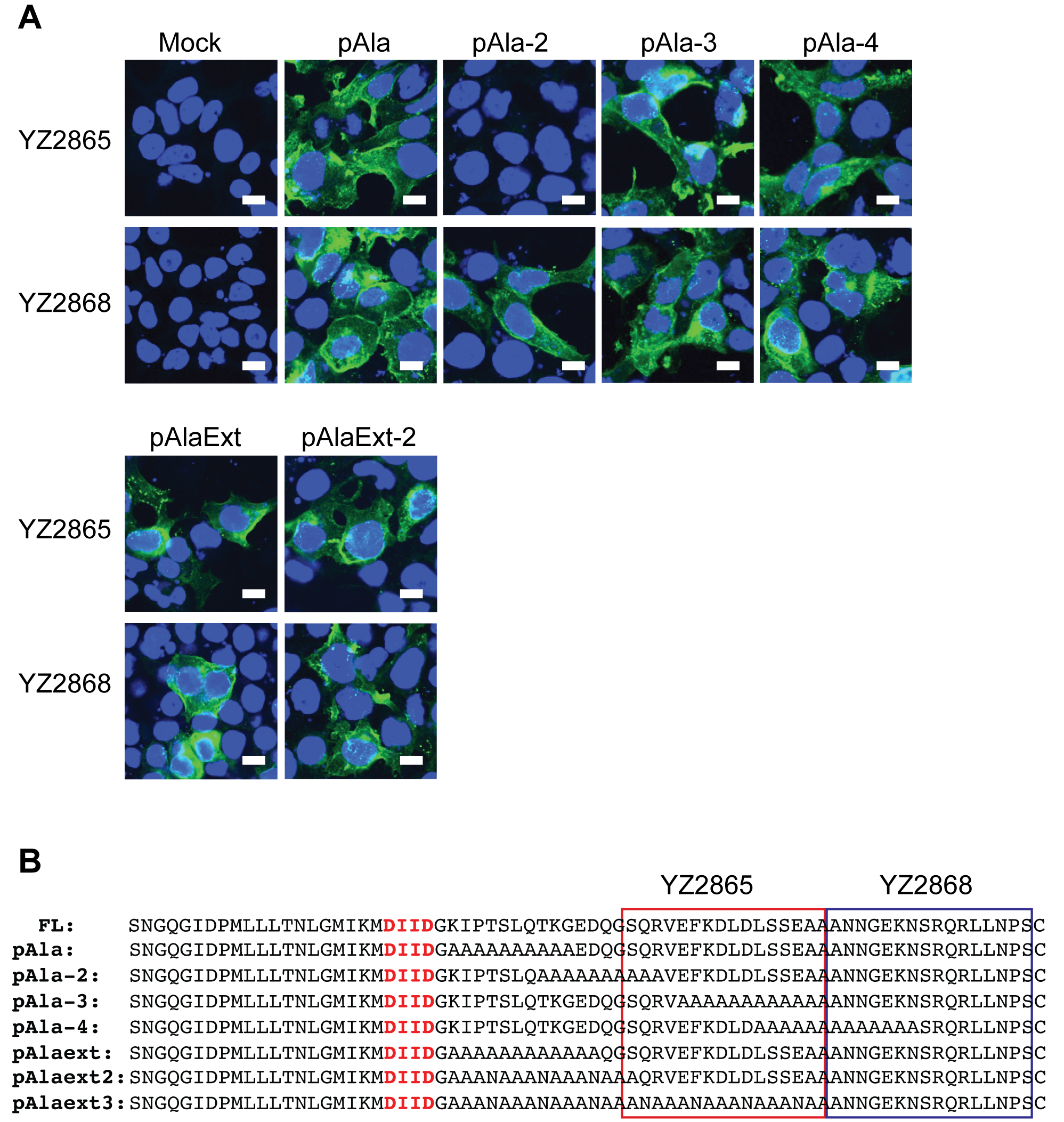

Supplement: Figure S6 — Staining of cells expressing Panx1 poly-alanine mutants by immunocytochemistry. (A) HEK293T cells expressing one of the various inactive pAla mutants (pAla, pAla-2, pAla-3, pAla-4, pAlaExt, pAlaExt-2) were stained with two polyclonal antibodies with distinct c-terminal epitopes, YZ2865 and YZ2686. Both antibodies were able to stain all of the pAla mutants with the exception of pAla-2, which showed no staining with YZ2865 likely due to disruption of the epitope. In the case of each of these mutants, staining appeared consistent with membrane expression. Scale bars for images are 10 µm. (B) The sequences of the mutants tested as well as the location of the peptides used to generate the antibodies are shown. (TIF) [file pone.0099596.s006.tif]

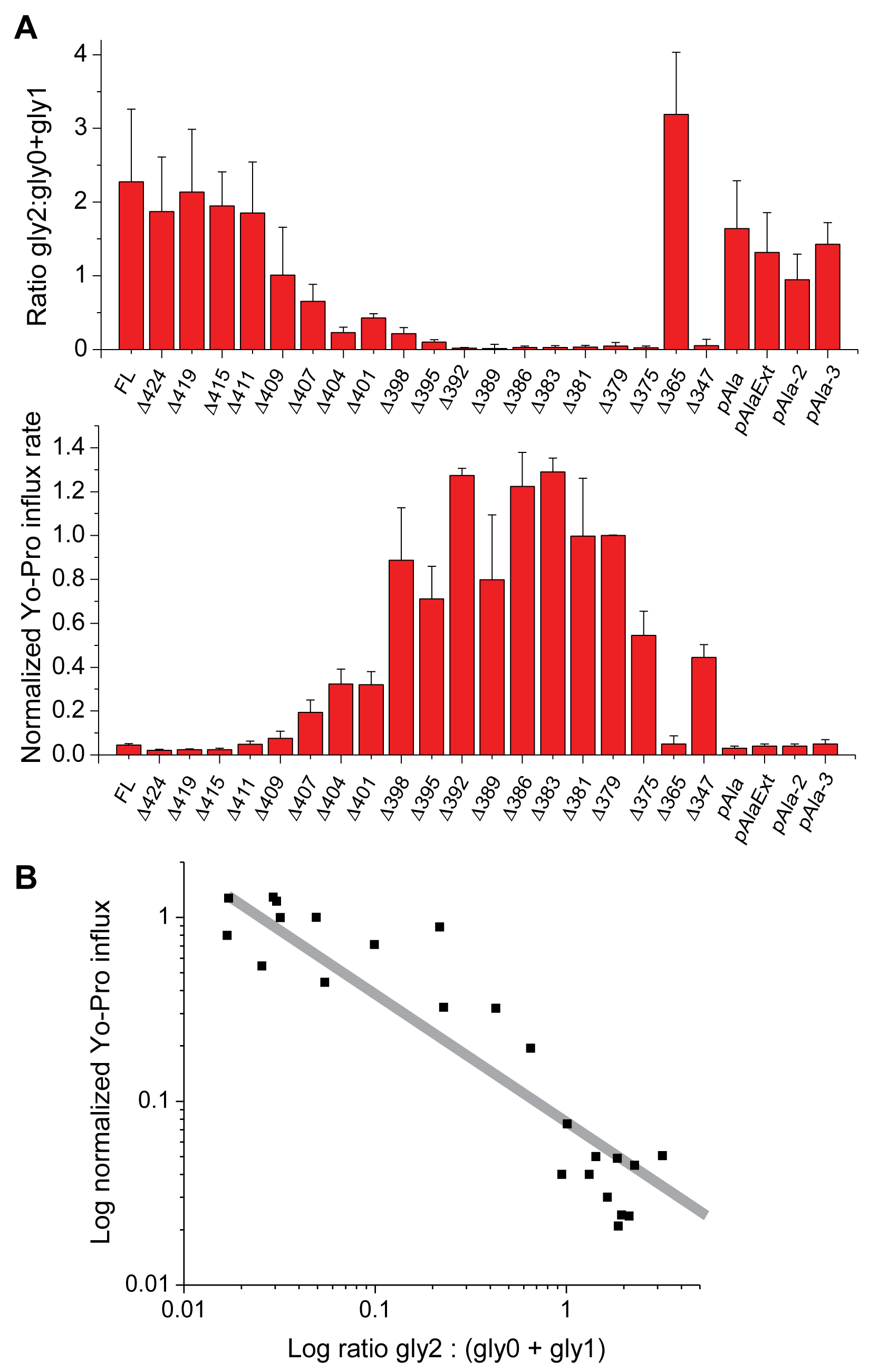

Supplement: Figure S7 — The absence of the heavily-glycosylated form of Panx1 (gly2) correlates with constitutive functional activity. To quantify the amount of gly2 form present when the various Panx1 mutants were expressed in this study, we measured the density of the gly0, gly1, and gly2 bands from Western blots of the surface fractions. (A) We calculated the ratio gly2: (gly0+gly1) for each mutant and averaged together data from 2–3 Western blots per mutant. Error bars represent SEMs calculated for each mutant. (A-below) Plotted along side are the normalized Yo-Pro influx rates measured for each of these mutants. In general, mutants with high Yo-Pro influx rates have low levels of gly2, whereas mutants with low Yo-Pro influx rates have high levels of gly2 relative to gly0 and gly1. (B) To further examine this correlation, we plotted the log of the normalized Yo-Pro influx rate against the log of the gly2:(gly0+gly1) ratio. The line shown in grey shows the overall trend in the data but in no way implies that this relationship should be linear. To determine whether this apparent correlation is significant, we calculated the Spearman's Rank Correlation Coefficient, which determines whether a correlation exists in a non-parametric way without any assumption as to the functional form of this correlation. In this case, we find that the Spearman's Rank Correlation Coefficient = −0.871 indicating strong negative correlation. (TIF) [file pone.0099596.s007.tif]

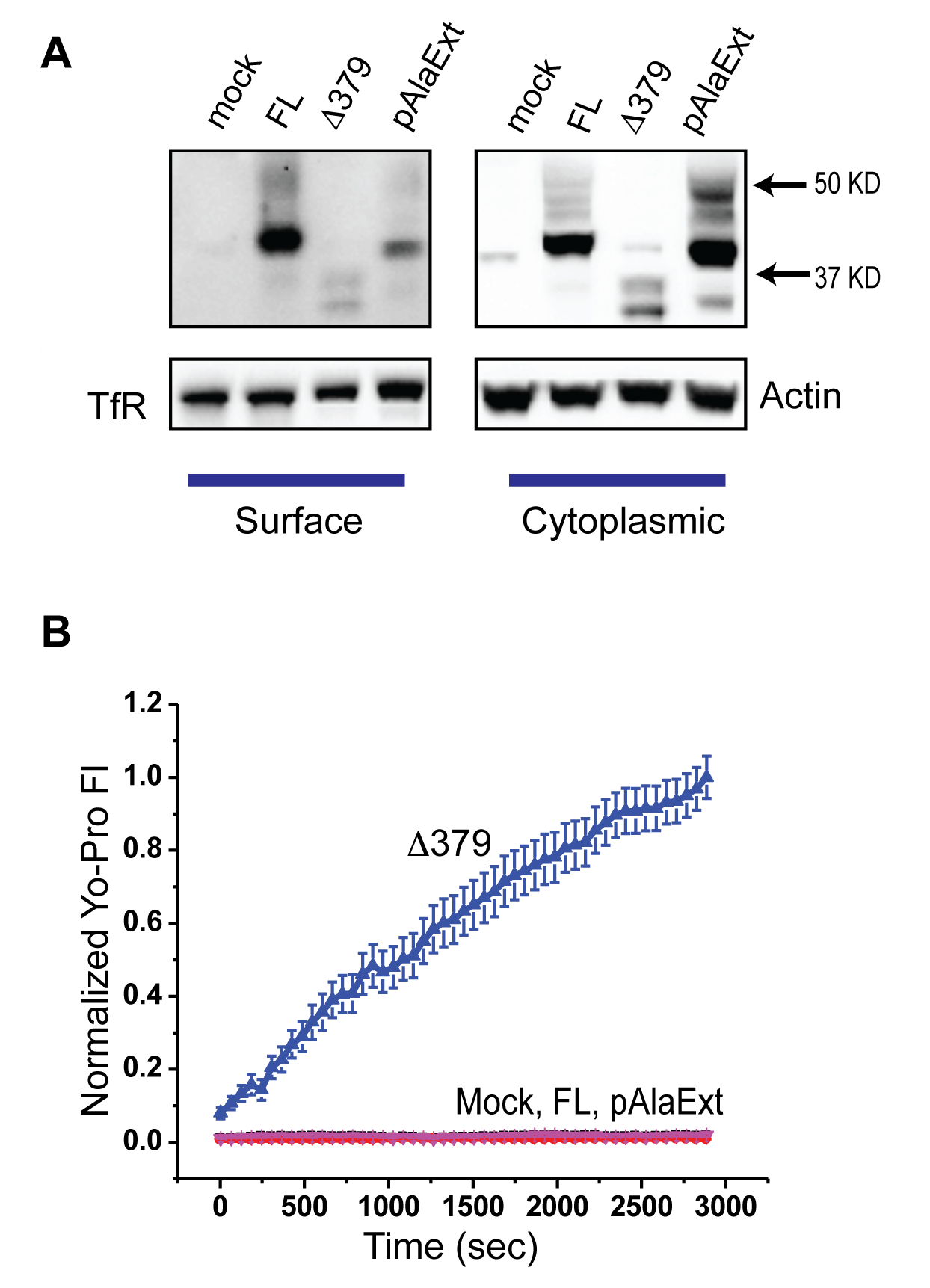

Supplement: Figure S8 — Human Panx1 (Δ379), but not human full-length or pAlaExt, is constitutively active. Human FL Panx1 as well as human mutants Δ379 and pAlaExt and pCDNA3.1 vector-only (mock) were transiently expressed in HEK293T cells. (A) Surface biotinylation- Western blots show that the FL human Panx1 channel as well as the human Δ379 and pAlaExt mutants are expressed in the membrane surface at very similar levels to the equivalent mPanx1 constructs. (B) As in the case of the equivalent mouse Panx1 channel, human Δ379 showed robust Yo-Pro influx following addition of 1 µg/mL Yo-Pro-1 while human FL and pAlaExt showed no detectable Yo-Pro influx after 50 min, similar to vector-only transfected cells. (TIF) [file pone.0099596.s008.tif]

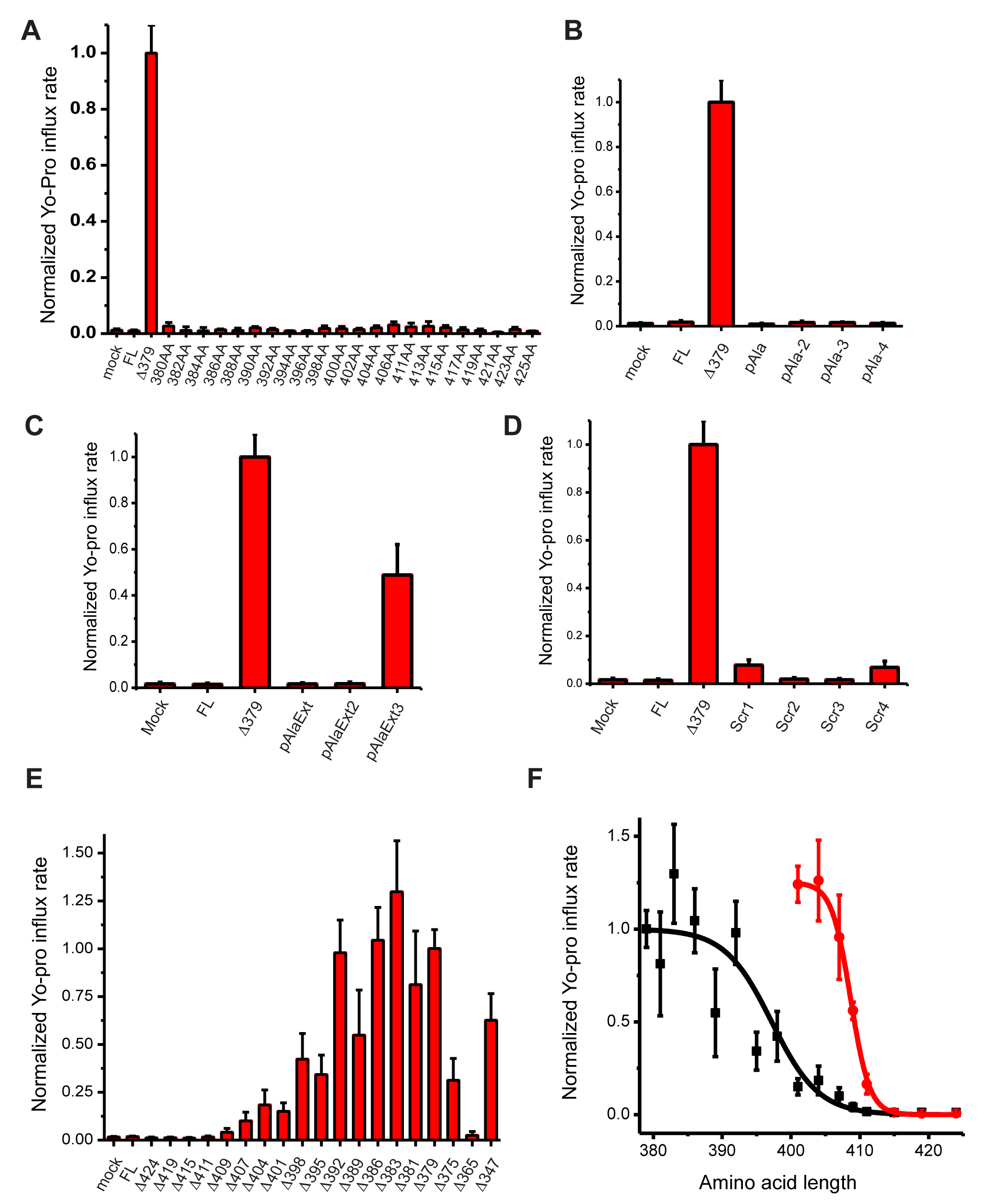

Supplement: Figure S9 — Re-analysis of Yo-Pro influx data while correcting for surface expression differences. The Yo-Pro influx rate data collected for each Panx1 mutant was corrected to account for differences in surface expression level of each mutant. Briefly, we divided Yo-Pro influx rate by relative surface expression level to obtain a corrected Yo-Pro influx rate. The new error bars represent SEM corrected to account for propagation of error. Thus, while the data have been re-normalized to the Δ379 mutant, the Δ379 mutant now has a measurable SEM resulting from the error in the measurement of expression level. Shown here are (A) corrected and re-normalized Yo-Pro influx rate for the double-alanine mutants, (B) the pAla mutants, (C) the pAlaExt mutants, (D) the scrambled mutants, and (E) the truncation mutants. (F) The presence of the pAlaExt mutation shifted the expression-corrected function-vs-length-dependence of c-terminal truncations by 11.5 amino acids, as determined by fitting both sets of truncation mutant data to a 3-parameter logistic function. The parameters were obtained as follows: for the wild-type channel, max = 1.0, xc = 397.2, and slope factor = −0.293. For the pAlaExt mutant channel, max = 1.25, xc = 408.6, and slope factor(k) = −0.72. The logistic function used was y = max/(1+exp(−k*(x-xc))). (TIF) [file pone.0099596.s009.tif]
