# Supplementary material for: Pannexin-1 Is Blocked by Its C-Terminus through a Delocalized Non-Specific Interaction Surface
Source: PLoS One. 2014 Jun 9;9(6):e99596. doi: 10.1371/journal.pone.0099596 (PMC4049774; doi:10.1371/journal.pone.0099596)
Supplement: Table S1 — Surface expression and constitutive activity of Panx1 mutants examined in this study. Membrane surface expression (as determined by analysis of biotinylation-pull down Western blots) and Yo-Pro influx rate for each mutant Panx1 channel are shown. Results are presented as mean ± standard error of the mean (SEM). (DOC) [file pone.0099596.s010.doc]

**Table S1: Surface expression of Panx1 mutants examined in this study**

| **Mutation name** | **Surface expression** | **Yo-pro influx rate** |
| --- | --- | --- |
| Panx1 FL | 1.00 ± 0.00 | 0.04 ± 0.01 |
| Δ424 | 0.71 ± 0.15 | 0.02 ±0.01 |
| Δ419 | 0.76 ± 0.19 | 0.02 ± 0.00 |
| Δ415 | 0.89 ± 0.11 | 0.02 ± 0.01 |
| Δ411 | 1.21 ± 0.23 | 0.05 ± 0.01 |
| Δ409 | 0.73 ± 0.16 | 0.08 ± 0.03 |
| Δ407 | 0.78 ± 0.26 | 0.19 ± 0.06 |
| Δ404 | 0.71 ± 0.26 | 0.32 ± 0.07 |
| Δ401 | 0.86 ± 0.20 | 0.32 ± 0.06 |
| Δ398 | 0.85 ± 0.14 | 0.89 ± 0.24 |
| Δ395 | 0.84 ± 0.18 | 0.71 ± 0.15 |
| Δ392 | 0.52 ± 0.09 | 1.27 ± 0.03 |
| Δ389 | 0.59 ± 0.13 | 0.80 ± 0.29 |
| Δ386 | 0.47 ± 0.05 | 1.22 ± 0.15 |
| Δ383 | 0.40 ± 0.08 | 1.29 ± 0.06 |
| Δ381 | 0.49 ± 0.11 | 1.00 ± 0.26 |
| Δ379 | 0.40 ± 0.04 | 1.00 ± 0.00 |
| Δ375 | 0.70 ± 0.21 | 0.54 ± 0.11 |
| Δ365 | 0.80 ± 0.16 | 0.05 ± 0.04 |
| Δ347 | 0.29 ± 0.05 | 0.44 ± 0.06 |
| Δ327 | 0.00 ± 0.00 | 0.02 ± 0.01 |
| 425AA | 1.23 ± 0.17 | 0.03 ± 0.00 |
| 423AA | 1.08 ± 0.18 | 0.04 ± 0.02 |
| 421AA | 0.99 ± 0.08 | 0.01 ± 0.00 |
| 419AA | 1.17 ± 0.12 | 0.03 ± 0.01 |
| 417AA | 1.08 ± 0.12 | 0.04 ± 0.02 |
| 415AA | 0.97 ± 0.16 | 0.05 ± 0.02 |
| 413AA | 0.94 ± 0.21 | 0.06 ± 0.04 |
| 411AA | 1.13 ± 0.14 | 0.07 ± 0.04 |
| 406AA | 0.94 ± 0.08 | 0.07 ± 0.03 |
| 404AA | 1.12 ± 0.19 | 0.06 ± 0.02 |
| 402AA | 1.23 ± 0.13 | 0.04 ± 0.01 |
| 400AA | 1.13 ± 0.16 | 0.05 ± 0.02 |
| 398AA | 0.90 ± 0.25 | 0.04 ± 0.02 |
| 396AA | 1.11 ± 0.23 | 0.02 ± 0.00 |
| 394AA | 1.29 ± 0.06 | 0.03 ± 0.00 |

| **Mutation name** | **Surface expression** | **Yo-pro influx rate** |
| --- | --- | --- |
| 392AA | 1.14 ± 0.09 | 0.04 ± 0.01 |
| 390AA | 1.00 ± 0.13 | 0.05 ± 0.01 |
| 388AA | 1.19 ± 0.12 | 0.03 ± 0.02 |
| 386AA | 1.12 ± 0.18 | 0.04 ± 0.00 |
| 384AA | 1.00 ± 0.29 | 0.03 ± 0.03 |
| 382AA | 0.81 ± 0.11 | 0.02 ± 0.02 |
| 380AA | 0.99 ± 0.25 | 0.07 ± 0.03 |
| pAla | 1.14 ± 0.15 | 0.03 ± 0.01 |
| pAla-2 | 0.85 ± 0.17 | 0.04 ± 0.01 |
| pAla-3 | 1.07 ± 0.12 | 0.05 ± 0.02 |
| pAla-4 | 0.98 ± 0.10 | 0.03 ± 0.01 |
| pAla-5 | 0.02 ± 0.01 | 0.02 ± 0.01 |
| pAlaExt | 0.97 ± 0.11 | 0.04 ± 0.01 |
| pAlaExt2 | 0.84 ± 0.12 | 0.04 ± 0.01 |
| pAlaExt3 | 0.65 ± 0.10 | 0.79 ± 0.17 |
| pAlaExt-Δ424 | 1.07 ± 0.07 | 0.01 ± 0.00 |
| pAlaExt-Δ419 | 1.02 ± 0.09 | 0.01 ± 0.00 |
| pAlaExtΔ415 | 0.98 ± 0.06 | 0.01 ± 0.00 |
| pAlaExtΔ411 | 1.32 ± 0.11 | 0.16 ± 0.05 |
| pAlaExtΔ409 | 0.87 ± 0.07 | 0.56 ± 0.05 |
| pAlaExtΔ407 | 0.49 ± 0.05 | 0.96 ± 0.23 |
| pAlaExtΔ404 | 0.45 ± 0.06 | 1.26 ± 0.22 |
| pAlaExtΔ401 | 0.44 ± 0.02 | 1.24 ± 0.10 |
| Scr1 | 1.37 ± 0.20 | 0.26 ± 0.06 |
| Scr2 | 0.84 ± 0.15 | 0.04 ± 0.01 |
| Scr3 | 0.81 ± 0.21 | 0.03 ± 0.01 |
| Scr4 | 2.39 ± 0.51 | 0.41 ± 0.12 |
|  |  |  |
|  |  |  |
|  |  |  |
|  |  |  |
|  |  |  |
|  |  |  |
|  |  |  |
|  |  |  |
